# Light-Activated Qubit Coupling in a Vanadyl Porphyrin Trimer

**DOI:** 10.1021/jacs.5c17205

**Published:** 2026-03-06

**Authors:** Alberto Privitera, Alessandro Chiesa, Fabio Santanni, Davide Ranieri, Prem P. Sahu, Matthew D. Krzyaniak, Andrea Caneschi, Ryan M. Young, Mathias O. Senge, Federico Totti, Michael R. Wasielewski, Stefano Carretta, Roberta Sessoli

**Affiliations:** † Department of Industrial Engineering, 9300 University of Florence & UdR INSTM Firenze, Firenze 50121, Italy; ‡ Department of Chemistry, Center for Molecular Quantum Transduction, and Institute for Quantum Information Research and Engineering, 3270 Northwestern University, Evanston, Illinois 60208-3113, United States; § Department of Mathematical, Physical and Computer Sciences, 9370 University of Parma & UdR INSTM, Parma 43124, Italy; ∥ Department of Chemistry “U. Schiff”, 9376 University of Florence & UdR INSTM Firenze, Sesto Fiorentino 50019, Italy; ⊥ School of Chemistry, Trinity Biomedical Sciences Institute, Trinity College Dublin, 8809 The University of Dublin, Dublin D02R590, Ireland

## Abstract

Molecules provide a modular and chemically tunable platform for quantum information science. In recent years, significant advances have been made in enabling optical spin initialization, coherent control, and both optical and electrical readout of molecular qubits. Yet, a central challenge remains: realizing scalable architectures through the controlled and ultrafast activation of interqubit interactions. Here, we present a molecular system composed of two vanadyl porphyrin qubits bridged by a free-base porphyrin chromophore, where the qubits are magnetically independent in the ground state but become coupled upon photoexcitation. Femtosecond transient absorption and time-resolved electron paramagnetic resonance experiments, supported by DFT calculations and spectral simulations, reveal that photoexcitation induces the formation of a spin-quintet state within subpicosecond time scales. Notably, long-lived spin polarization persists up to room temperature. Theoretical modeling offers design principles for harnessing this mechanism in future applications. These results provide a proof of concept for optically controlled spin interactions in molecules, paving the way for light-activated molecular quantum gates.

## Graphical abstract

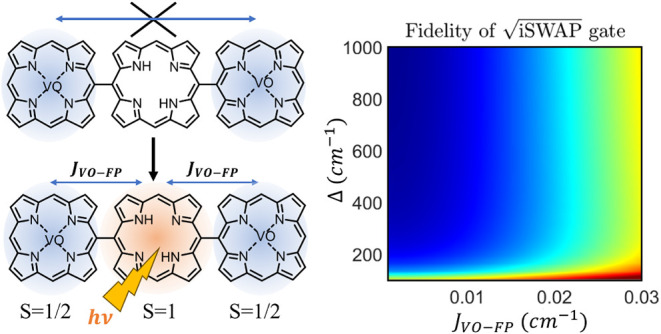

## Introduction

Electron spins are widely explored as qubit candidates for applications in quantum computing,^1,2^ sensing,^3–5^ and transduction.^6,7^ So far, the most extensively studied electron spin qubits have been realized through top-down fabrication approaches,^8^ such as nitrogen-vacancy (NV) centers in diamond^9,10^ and semiconductor quantum dots.^11–13^ In contrast, molecular systems offer a bottom-up alternative that brings unique advantages to quantum information science (QIS).^14–16^ These systems enable atomic-level control over electronic and magnetic properties and feature intrinsic structural reproducibility that facilitates qubit fabrication.^17–19^ Among them, transition metal complexes stand out due to the tunability of their spin Hamiltonian—allowing precise control over spin multiplicity, g-tensor, and hyperfine anisotropies. Their chemical stability, compatibility with surface processing, and modular architecture make them particularly attractive for integration into solid-state quantum devices.^20–23^

Nevertheless, a key challenge in molecular QIS remains unresolved: how to construct modular qubit systems that allow both single-qubit control and the selective activation of qubit–qubit interactions required for entangling quantum operations. Most efforts to date have focused on designing molecular architectures with spin centers weakly and permanently interacting through dipolar coupling or superexchange.^24–30^ However, permanent couplings pose fundamental limitations. They can lead to uncontrolled many-body dynamics and hinder gate implementation unless complex correction schemes are introduced.^29^ For scalable and flexible quantum architectures, it is crucial to develop qubit–qubit couplings that can be externally switched on demand. Various molecular strategies have been proposed to address this issue. These include redox-active bridges that modulate exchange coupling in response to external stimuli,^31^ as well as complexes exhibiting spin-crossover transitions or redox isomerism.^32,33^ While promising, such approaches often suffer from slow switching kinetics and limited functionality across broad temperature ranges and different environments.

An attractive alternative is to use light to trigger interactions between molecular qubits selectively. Photoexcited spin-bearing states have garnered increasing attention for quantum applications, including spin-triplet qubits,^34–37^ spin-correlated radical pairs,^38–42^ and chromophore-radical systems.^43–49^ Magnetic photoexcited states offer a pathway to fast, controllable coupling over a broad temperature range—provided that they adequately couple with the otherwise noninteracting qubits and the photoexcited spin state is sufficiently long-lived to perform the two-qubit logic operation. Furthermore, many photoexcited states exhibit non-Boltzmann spin populations, even at room temperature, potentially increasing their efficiency as quantum sensors.^50,51^

Among light-driven molecular systems based on transition metals,^52–55^ the chromophore-qubit architecture is attractive due to its high modularity, enabling independent optimization over the photophysical and spin properties of the chromophore and the qubit.^18,43^ A representative example of this class of compounds is the free base porphyrin chromophore covalently attached to a vanadyl porphyrin qubit.^56^ Upon selective excitation of the chromophore, it was observed that a triplet state (*S*  = 1) forms within a few picoseconds and couples with the spin-doublet of the vanadyl (*S*  = 1/2), generating a spin-polarized quartet state (*S * = 3/2) that can be detected by time-resolved electron paramagnetic resonance (EPR) spectroscopy, even at room temperature. Motivated by these results, we sought to explore whether the photoexcited triplet state could mediate an exchange interaction between two molecular qubits that are magnetically decoupled in the ground state. In contrast to other mechanisms where light controls exchange interactions through photoreactions of the bridge, here the photoexcitation leads to the formation of a *S* > 0 state on the bridge. This activates an effective coupling between the terminal spins, which is then reversibly switched off when the bridge relaxes back to the ground state. To date, only a limited number of ground-state systems comprising two *S*  = 1/2 centers bridged by a chromophore have been reported, and these are based on organic radicals.^57–69^ Demonstrating the formation of a long-lived quintet state in a system based on transition metal qubits would provide a first proof-of-concept toward using photoexcited high-spin states to control magnetic coupling between otherwise noninteracting, highly tunable qubits.

In this work, we report the synthesis, photophysical properties, and spin polarization dynamics of a vanadyl porphyrin trimer, 5,15-[oxo­(10,20-diphenylporphyrin-5-ylato)­vanadium­(IV)]-10,20-diphenylporphyrin (**VO-FP-VO**). The molecular architecture consists of two [VO­(DPP)] (DPP^2–^ = 5–15-diphenylporphyrinate) units—each acting as a spin-doublet molecular qubit—covalently linked through a central diphenylporphyrin chromophore. In the ground state, the echo-detected EPR (EDEPR) spectra at X- and W-bands are indistinguishable from those of isolated vanadyl porphyrins. Upon optical excitation, a triplet state (*S*  = 1) is formed, and time-resolved electron paramagnetic resonance (TREPR) spectroscopy reveals a net emissive signal up to room temperature. Orientation-dependent TREPR measurements, supported by spectral simulations and state-of-the-art DFT calculations, indicate the formation of a photoexcited quintet state (*S*  = 2), resulting from ferromagnetic coupling between the triplet chromophore and the two vanadyl spins. Theoretical modeling of the system provides molecular design guidelines for employing similar architectures as light-activated $\sqrt{iSWAP}$ gates offering a pathway toward scalable and switchable quantum architectures.

## Experimental Section

### Sample Preparation

Details on sample synthesis and characterization are provided in the text and in Section 1 of the Supporting Information (Figures S1–S3).^70–73^ Room-temperature femtosecond and nanosecond transient absorption (fsTA and nsTA) experiments were performed in toluene solutions prepared in 2 mm path length glass cuvettes and degassed by three freeze–pump–thaw cycles (10^–3^ Torr). Sample concentrations were adjusted to achieve an optical density (O.D.) of 0.3–0.6 at the excitation wavelength (∼100 μM). EPR samples were prepared using concentrations comparable to those employed in the TA experiments. For X-band measurements, solutions (∼100 μL) were loaded into quartz tubes (2.40 mm o.d., 2.00 mm i.d.), degassed via three freeze–pump–thaw cycles, and sealed under vacuum with a hydrogen torch. For low-temperature EPR experiments, samples were prefrozen in liquid nitrogen prior to insertion into the precooled resonator at 85 K. For W-band measurements, solutions were loaded into quartz tubes (0.86 mm o.d., 0.60 mm i.d.) and inserted directly into the cold resonator precooled to 85 K.

### Optical Spectroscopy

Steady-state absorption spectra were acquired on a Shimadzu 1800 spectrophotometer. The fsTA and nsTA experiments were conducted using a previously described instrument.^74,75^ Both measurements were performed by using a regeneratively amplified Ti:sapphire laser system operating at 1 kHz repetition rate to generate 828 nm pulses, which create the 550 nm excitation pulses using a commercial collinear optical parametric amplifier (TOPAS-Prime, Light Conversion, LLC). The TA spectra were acquired by using an excitation energy of about 1 μJ/pulse, a full-width half-maximum diameter (fwhm) ∼300 μm. The data were background-subtracted and chirp-corrected using a lab-written MATLAB program. The TA data were subjected to global kinetic analysis to obtain the evolution-associated and kinetic parameters as described in detail previously.^76^ The three-step model discussed in the text is the simplest model that provides a good and robust fit to the kinetic traces.

### Electron Paramagnetic Resonance (EPR) Spectroscopy

X- and W-band EPR measurements were performed using Bruker Elexsys E580 and E680 spectrometers, respectively. The E580 system was equipped with a split-ring resonator (Bruker ER4118X-MS3), while the E680 was equipped with a cylindrical resonator (Bruker EN-680-1021H) and a 2W solid-state amplifier. Temperature was controlled using an Oxford Instruments CF935 continuous-flow cryostat cooled with liquid nitrogen and an ITC503S temperature controller.

For X-band Time-Resolved Continuous-Wave EPR (TREPR) studies, the sample was photoexcited at 550 nm with 7 ns pulses generated via an optical parametric oscillator (GWU BasiScan) pumped with the 355 nm output of a frequency-tripled Nd:YAG laser (Spectra-Physics Quanta-Ray Lab-150–10H) operating at a repetition rate of 10 Hz. The laser light was coupled into the resonator via an optical fiber and a collimator positioned outside the cryostat window, delivering approximately 1 mJ per pulse with a fwhm beam diameter of ∼5 mm. Following photoexcitation, transient magnetization time traces were recorded as a function of magnetic field using direct diode detection under continuous microwave irradiation. The number of signal averages depended on temperature, typically 32 at 85 K and 128 at room temperature. The data were processed by first subtracting the background signal prior to the laser pulse for each kinetic trace (at a given magnetic field), then subtracting the signal at off-resonance magnetic fields for each spectrum (at any given time).

Echo-detected EPR (EDEPR) spectra were recorded using the standard two-pulse Hahn echo sequence (π/2 – τ – π). At W-band, Gaussian-shaped pulses were generated using an arbitrary waveform generator (AWG). Pulse lengths of π/2 = 16 ns and π = 32 ns were used at X-band, while π/2 = 20 ns and π = 40 ns were used at W-band.

### Spectral Simulations

EPR simulations were performed using a home-built code developed in the MATLAB scripting environment, supplemented by routines from the EasySpin simulation package.^77^ Further details of the spectral simulations are provided in the text.

### DFT Characterization

All quantum chemical calculations were performed using the ORCA 6.0.0 software suite.^78^

Full geometry optimizations were carried out using the B3LYP functional^79,80^ with the def2-SVP basis set^81^ on hydrogens and with larger def2-TZVP basis sets for all the other atoms.^81,82^ Dispersion effects were included via Grimme’s D3BJ correction,^83,84^ and implicit solvation was modeled using the CPCM model with toluene as the solvent.^85^ Harmonic frequency calculations confirmed that each optimized geometry corresponds to a true minimum on the potential energy surface.

To compute the exchange interaction with the ^3*^FP, we successfully applied the Broken Symmetry (BS) approach previously developed for the ground state and validated for very weak exchange regimes,^86,87^ to the photoexcited state. The isotropic exchange parameter between the vanadyl centers in the ground state, $J_{VO_{1}VO_{2}}$ , was calculated within the full projected formula:

$${Ĥ}_{ex}=J_{VO_{1}VO_{2}}{ŝ}_{VO_{1}}\cdot {ŝ}_{VO_{2}}$$ 1

2 $$J_{VO_{1}VO_{2}}=\frac{\left[E\left(HS\right)-E\left(BS\right)\right]}{2s_{VO_{1}}\cdot s_{VO_{2}}}$$

where the energy of the high spin state (HS) is that of *M*_*s*_ = $m_{{VO}_{1}}+m_{{VO}_{2}}$ = 1, while *E*(*BS*) = *E*(*M*_*s*_ = $m_{{VO}_{1}}+m_{{VO}_{2}}$ = 0). Here, $s_{VO_{x}}$ (with *x* = 1,2) correspond to 1/2.

The following spin Hamiltonian was used for the system where the chromophore is photoexcited in the *S*_*FP*_ = 1 state (Figure S6):

H^ex=JVO1VO2ŝVO1·ŝVO2+JVO1FPŝVO1·ŜFP+JVO2FPŝVO2·ŜFP 3

In this case, the three *J* were obtained by computing the following BS determinants using the basis $\left|m_{{VO}_{1}}m_{{VO}_{2}}m_{FP}\right\rangle$ : |1/2, 1/2, 1>; (HS, *m*_*s*_ = 2), |-1/2, 1/2, 1>; (BS1, *m*_*s*_ = 1), and |1/2,‐1/2,1⟩ (BS2, *m*_*s*_ = 1), and |1/2, 1/2, -1> (BS3, *m*_*s*_ = 0).

The *m*_*FP*_ = ±1 states of ^3*^FP were constructed by driving the calculations to converge on the TD-DFT excitation corresponding to the HOMO→LUMO transition of FP. This excitation provides the main contribution to the computed transitions at 551 and 595 nm (see Figure S9), which have a high oscillator strength. The transition at 551 was chosen as the best guess in agreement with the experimental results. We employed the same CAM-B3LYP functional and the basis sets scheme used for the ground state.^88^

BS states for the ground and excited states were calculated at the CAM-B3LYP and B3LYP level to check the effect of the long-range interactions and to have a direct comparison with the *J* values already reported in the literature for the VO dimers.^26^ The computed differences are very small and, therefore, only the B3LYP values will be used in the main text, while the CAM-B3LYP ones can be found in the SI. The orbitals involved and the spin densities computed for the different BS states in the ground and the excited state are reported in SI (Figures S5, S7, and S8). A strict VeryTIGHTSCF convergence criterion (SCFCONV10), corresponding to an energy change threshold of 1.0 × 10^–10^ hartree, was employed thoroughly, and no convergence accelerators or auxiliary basis sets were used. Full computational details and data are provided in the Supporting Information.

## Results and Discussion

### Synthesis and Ground-State Properties

**VO-FP-VO** was synthesized following the general scheme reported in Figure 1a using a stepwise convergent synthetic approach.^89,90^ In previous works,^24,26,56^ we reported that Suzuki coupling between an iodo-substituted metalloporphyrin and a free base porphyrin provides an efficient route to heterometallic or hybrid metalloporphyrin/free base porphyrin systems. Here, we applied a similar strategy using [VO­(DPPI)] (DPPI^2–^ = 5-iodo-10,20-diphenylporphyrinate) and 5,15-diphenyl-10,20-bis­(4,4,5,5-tetramethyl-1,3,2-dioxaborolan-2-yl)­porphyrin (H_2_DPPBPin_2_) as precursors.^71,91^ The reaction yields a mixture of oligomers ranging from dimers to tetramers, typically separated by column or size-exclusion chromatography.^92,93^ In this case, the desired trimer was isolated by silica gel chromatography, as confirmed by the characterization data (Figures S1–S3).

**Figure 1:**
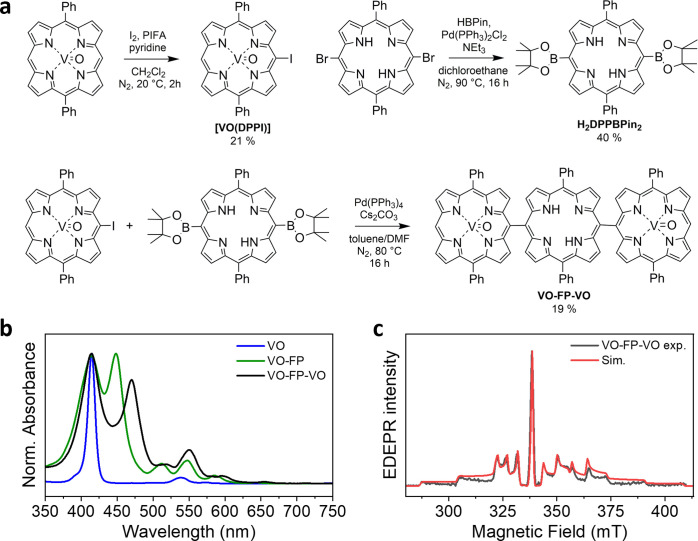
(a) Chemical structure and synthetic route of the vanadyl–free-base–vanadyl porphyrin trimer (**VO-FP-VO**). (b) Steady-state UV/vis absorption spectrum of **VO-FP-VO** in toluene at room temperature, compared with the previously reported spectra of the monomeric vanadyl porphyrin (**VO**) and the vanadyl–free-base porphyrin dimer (**VO-FP**). (c) X-band echo-detected EPR spectrum of **VO-FP-VO** (black trace) in toluene at 85 K, overlaid with the spectral simulation (red trace) performed using the same EPR parameters of VO: g = [1.985, 1.985, 1.964] and A­(^51^V) = [162, 162, 475] MHz.

The steady-state UV–vis absorption spectrum of **VO-FP-VO** in toluene at room temperature is shown in Figure 1b, alongside those of the previously reported oxo­(5,10,15-triphenylporphyrinato)­vanadium­(IV) (**VO**) and 5-[oxo­(10,20-diphenylporphyrinato-5-yl)­vanadium­(IV)]-10,20-diphenylporphyrin (**VO-FP**).^56^ As commonly observed for porphyrin derivatives, the spectra display two main features: intense Soret bands in the near-UV region (∼400–475 nm) and weaker Q-bands in the visible range (∼500–650 nm), both arising from π–π* transitions within the macrocycle.^94^ In porphyrin arrays, exciton coupling is strongly influenced by the length and geometry of the linkers connecting adjacent units.^95,96^ In **VO-FP-VO**, aside from a relatively small red shift, the Q-band region remains largely unperturbed relative to that of the monomer, indicating weak electronic coupling in the lowest singlet excited state (*S*_1_)—likely due to the near-orthogonal orientation of adjacent porphyrin units. By contrast, stronger excitonic interactions are evident in the *S*_2_ state, as indicated by the splitting of the Soret bands. Such splitting reflects coupling between porphyrin units and is characteristic of directly linked meso–meso arrays, where exciton coupling energies have been reported to reach ∼2100 cm^–1^.^96^

The X-band and W-band EDEPR spectra of **VO-FP-VO** in frozen toluene at 85 K (black traces) are shown in Figures 1c and S10, respectively, alongside spectral simulations (red traces). Both spectra exhibit the characteristic powder pattern of vanadyl porphyrins, dominated by the anisotropic hyperfine interaction between the electron spin and the ^51^V nucleus (*I* = 7/2).^26,97,98^ The simulations were performed using standard parameters for vanadyl porphyrin monomers (g = [1.985, 1.985, 1.964] and A­(^51^V) = [162, 162, 475] MHz). Notably, no discernible spectral features attributable to intervanadyl coupling are observed in the multifrequency analysis, suggesting that any exchange or dipolar interaction lies below the inhomogeneous line width. The phase memory time, *T*_*m*_, of VO-FP-VO in toluene was also measured at W-band at 85 K (Figure S11) and provided values consistent with previous reports on VOTPP.^24,26^

### Transient Absorption Spectroscopy

Femtosecond/nanosecond transient absorption (TA) spectroscopy was performed on **VO-FP-VO** in deoxygenated toluene at room temperature upon 550 nm photoexcitation (Figure 2a). This excitation wavelength was chosen for consistency with EPR experiments (see below). Additionally, previous results on **VO-FP** showed no significant differences between 550 and 650 nm excitation.^56^ Global kinetic analysis provided evolution-associated spectra (EAS) with associated time constants for the formation and decay of intermediate species (Figure 2b–d).

**Figure 2:**
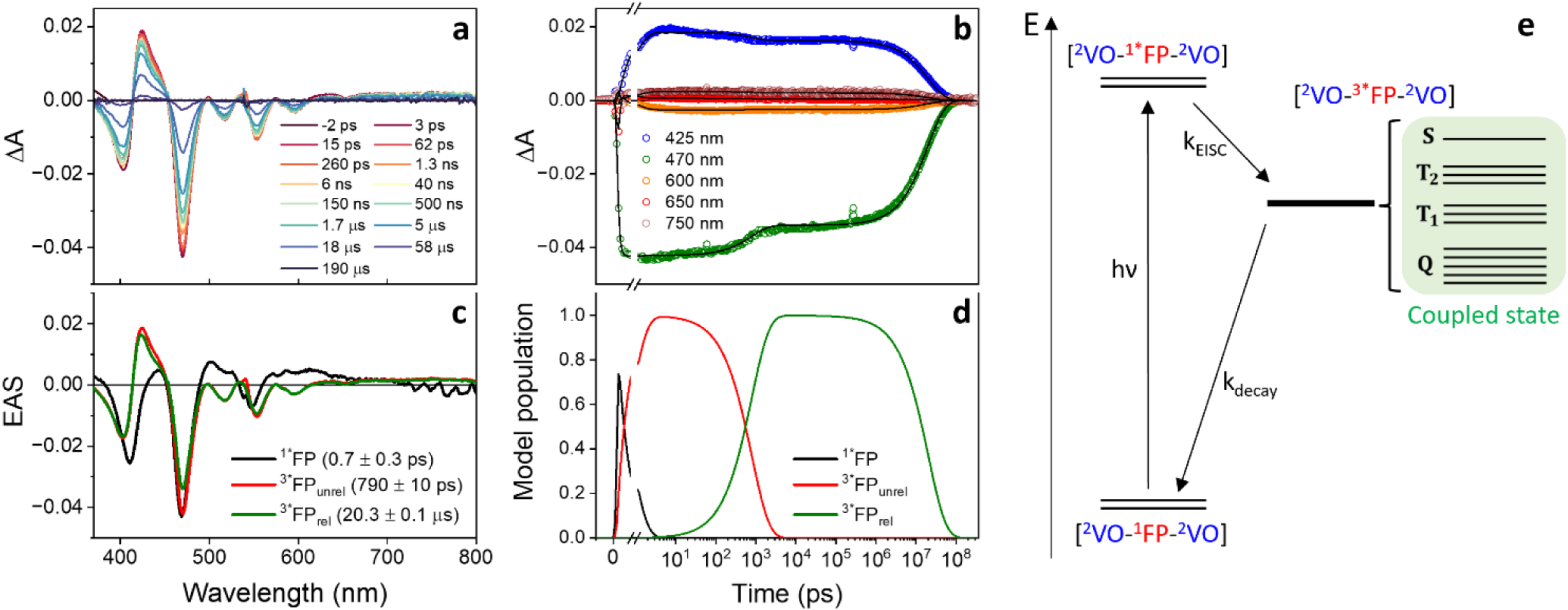
(a) Transient Absorption (TA) spectra of **VO-FP-VO** in toluene at room temperature, excited at 550 nm and recorded at selected delay times. (b) Kinetic traces at representative wavelengths with corresponding global fits. (c) Evolution-associated spectra (EAS) extracted from the global fit analysis. (d) Population dynamics based on a sequential kinetic model ^1*^FP → ^3*^FP_unrel_ → ^3*^FP_rel_ → ground state. In this model, state ^1*^FP corresponds to the singlet excited state of FP. States ^3*^FP_unrel_ and ^3*^FP_rel_ display similar spectral features but differ in kinetics, suggesting they arise from the same electronic state—the FP triplet—with state ^3*^FP_unrel_ assigned to an unrelaxed triplet configuration and state ^3*^FP_rel_ to its structurally relaxed form. (e) Schematic representation of the observed photophysical pathways in **VO-FP-VO**.

Immediately after excitation, the spectra display characteristic ground-state bleach (GSB) features corresponding to the Soret and Q bands, along with a broad excited-state absorption (ESA) spanning the visible region.^56,99^ These features are assigned to the singlet excited state of the free-base porphyrin moiety in the trimer (^2^VO-^1*^FP-^2^VO). At 550 nm, both VO and FP units are likely excited. However, only ^1*^FP is observed immediately after excitation due to ultrafast energy transfer from VO to FP within the ∼300 fs instrument response.^100,101^

The singlet excited state of the FP unit decays with a time constant of 0.7 ± 0.3 ps, leading to the formation of a long-lived triplet state localized on the porphyrin (^2^VO-^3*^FP-^2^VO).^56^ This intermediate subsequently evolves over 790 ± 10 ps into a second state with a similar EAS but distinct amplitude and kinetic behavior, indicating that both species share the same electronic configuration. We assign the initial species (^3*^FP_unrel_) to the unrelaxed triplet state and the latter (^3*^FP_rel_) to the structurally relaxed triplet, consistent with previous observations.^48^ The GSB and ESA features of the triplet excited state decay to the ground state with a time constant of 20.3 ± 0.1 μs.

A comparison of the kinetic time constants for ISC and triplet decay in **VO**, **VO-FP**, and **VO-FP-VO** is presented in Table S5. As previously reported, **VO** exhibits rapid ISC and triplet decay, driven by the close spatial proximity between the excited state of the porphyrin ligand and its paramagnetic vanadyl center.^102^ In **VO-FP**, both processes are slower, consistent with triplet localization on the adjacent diamagnetic free-base porphyrin.^56^ In **VO-FP-VO**, the rates are intermediate, reflecting a triplet localized on the FP unit, yet influenced by the magnetic interactions with two adjacent VO centers.

A schematic representation of the observed photophysical pathways is shown in Figure 2e. Following intersystem crossing, the transient absorption data are consistent with a FP-centered triplet state; however, they do not allow discrimination among the nearly degenerate singlet (S), triplet (T_1_ and T_2_), and quintet (Q) spin states that arise from exchange interaction with the two vanadyl centers in the strong coupling regime.^103^ This observation is consistent with previous experiments in chromophore-biradical systems,^63^ where closely spaced spin manifolds remain unresolved by TA spectroscopy.

### Exchange Coupling in the Photoexcited State

To gain further insight into the spin multiplicity of the photoexcited state in **VO-FP-VO**, we performed TREPR spectroscopy in frozen toluene at 85 K. Experiments were performed using both 550 and 650 nm excitation wavelengths (Figure S12), revealing no significant differences, as previously observed for VO-FP.^56^ Therefore, subsequent experiments were conducted using 550 nm excitation, which corresponds to a stronger absorption band and yields an improved signal-to-noise ratio.

The TREPR spectra in Figure S12 show a net emissive polarization in the 310–375 mT range, persisting beyond 7 μs—consistent with previous observations in **VO-FP**.^56^ In contrast to the dimer, no resolved hyperfine splitting from the ^51^V nucleus is observed in the trimer.

To investigate the degree of ordering of the nematic phase, we conducted orientation-dependent TREPR experiments using the nematic liquid crystal 4-cyano-4′-pentylbiphenyl (5CB) as the solvent and acquired spectra with the 5CB director oriented either parallel or perpendicular to the external magnetic field (***B_0_***). Based on previous studies, the molecule is expected to align its meso–meso axis (*y* direction, see Figure 3c for the reference frames) along the 5CB director.^104^ Consequently, the parallel orientation primarily probes the *g_VO,y_*/*A_VO,y_* components, while the perpendicular orientation probes the *g_VO,x_*/*A_VO,x_* and *g_VO,z_*/*A_VO,z_* components. This was verified by recording the EPR spectra of the ground state. The simulation reported in Figure S16 allowed the estimation of the width of the orientational distribution, resulting in σ = ±5°. This small degree of disorder is well accounted for by the line width used in the simulation of TREPR spectra.

**Figure 3:**
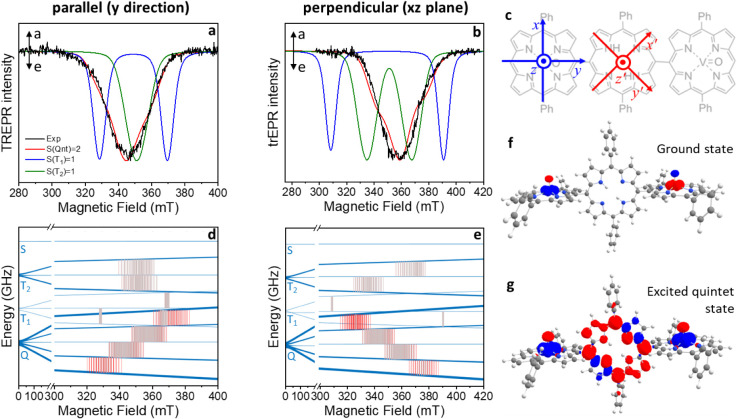
(a, b) Normalized 1D experimental TREPR spectra (black line) and spectral simulations (green, blue and red lines) of **VO-FP-VO** aligned in the nematic liquid crystal 5CB at 85 K, taken 1.5 μs after a 550 nm laser pulse (duration 7 ns, energy 2 mJ). The **VO-FP-VO** molecules are oriented with their long axis parallel (a) and perpendicular (b) to the external magnetic field direction. Simulation legend: blue line = population on T_1_ triplet state; green line = T_2_ triplet state; red line = quintet state, Q. (c) Orientation of the principal axes of the ^2^VO (blue) and ^3*^FP (red) units. The red and blue reference frames are rotated by 45° in the porphyrin plane, whereas a dihedral angle of 80° between the VO and FP planes (i.e., between *z* and *z*’) was assumed in the simulations. (d, e) Zeeman energy-level diagram and transitions of **VO-FP-VO** for two representative molecular orientations relative to the external magnetic field with the parameters reported in Table S6. Panel (d) corresponds to the molecular orientation probed in the parallel case (*y* || *B*_0_) and panel (e) the orientation *x* || *B*_0,_ which is one of the orientations probed in the perpendicular case. The color scale represents the transition amplitude, with red indicating allowed resonances and gray forbidden ones; transitions with relative intensity below 0.05 are omitted. The transitions inside each spin manifold correspond to the T_1_, T_2_, and quintet simulated signals shown in panels a and b. The following populations, in growing energy order, were assumed to compute the individual contributions to the spectra: T_1_ [0,0.33,0.67], T_2_ [0, 0.33, 0.67], Q [0, 0.09, 0.25, 0.33, 0.33]. (f, g) Spin densities (contour value of 0.015 e^–^/bohr^3^) computed for **VO-FP-VO** in the ground state (f) and the photoexcited quintet state (g), respectively.

TREPR spectra recorded at 1.5 μs after laser excitation in 5CB, are presented in Figure 3a,b, with the full time-resolved data sets provided in Figure S13. The TREPR spectra in 5CB exhibit a similar net emissive spin polarization to that observed in frozen toluene. The broader signal recorded for the perpendicular orientation compared to the parallel one is consistent with the larger *A*_VO,z_ component. Yet, the hyperfine pattern typically associated with vanadyl centers remains unresolved, even under alignment conditions, which prompted us to perform spectral simulations.

Due to the symmetry of the vanadyl *d*_*xy*_ orbital, vanadyl porphyrins can exhibit ferromagnetic exchange interactions both in the ground state when coupled to other paramagnetic centers^26,98^ or to an excited triplet state.^56,102,105^ These findings support the preliminary assignment of the TREPR signal to a long-lived photoexcited quintet state.

To validate this assignment, we performed DFT calculations on both the ground and photoexcited states to estimate the sign and magnitude of the exchange coupling and construct a spin energy level diagram for **VO-FP-VO**. The DFT investigation was conducted on the two plausible conformers of **VO-FP-VO**, which differ in the relative orientation of the vanadyl oxo ligands: “*cis*”(both VO moieties pointing in the same direction) and “*trans*”(pointing in opposite directions) (Figure S4). Geometry optimizations yielded stable structures with bond lengths and coordination geometries consistent with previous experimental and theoretical data on meso–meso vanadyl dimers (Table S1).^26^ The two conformers are nearly isoenergetic, differing by only 0.11 kcal·mol^–1^, suggesting they likely coexist under experimental conditions. The dihedral angles (θ and θ′) between the FP plane and each VO plane, defined as the mean plane of the four nitrogen atoms, are ∼89° and ∼91° (Figure S4). Notably, the rather shallow potential energy surface (PES) between θ = 60° and 90° indicates that multiple θ values are accessible, thereby giving rise to a conformational distribution. Indeed, crystallographic structures of meso–meso linked porphyrin units comprising a vanadyl unit present dihedral angles in the range 69°–93°.^24,26,98^ Computed magnetic exchange interactions in the ground state (Table S2) are practically null, $J_{VO_{1}VO_{2}}$ ­(*cis*) = −1.32 × 10^–5^ cm^–1^ and $J_{VO_{1}VO_{2}}$ ­(*trans*) = −3.95 × 10^–5^ cm^–1^ in the spin Hamiltonian formalism of eq 1. The corresponding BS orbitals (Figures 3f and S5) reveal that the spin density is highly localized on each vanadyl center, with minimal delocalization onto the FP unit. This localization arises from the lack of coplanarity between adjacent porphyrin rings, which restricts the overlap between the magnetic π-system of the VO units and the σ-framework of the FP ligand. Even for substantial deviations from orthogonality, the exchange interaction remains rather weak (∼1 × 10^–4^ cm^–1^ for θ = 60°), supporting the picture of two essentially noninteracting, magnetically isolated VO units in the ground state.

With the formation of the ^3^*FP excited state, new magnetic exchange pathways can arise both between the VO and FP units and between the two VO units. Excited-state HS/TD-DFT and BS/TD-DFT calculations consistently predict sizable ferromagnetic interactions between each VO and FP units (see Table S3), with exchange coupling constants on the order of 1 cm^–1^ $(J_{VO_{1}FP}$ = −0.72 cm^–1^ and $J_{VO_{2}FP}$ = −0.64 cm^–1^). The computed $J_{VO_{1}VO_{2}}^{*}$ value, 3.33 × 10^–2^ cm^–1^, confirms that direct VO-VO magnetic exchange is antiferromagnetic but remains weak even when FP is in the triplet excited state and no spin frustration arises. The ferromagnetic interactions between the VO and ^3*^FP can be rationalized on the same basis discussed before: the VO magnetic orbitals exhibit weak delocalization onto the σ-system of the FP unit, whereas the ^3*^FP shows pronounced delocalization onto both the π-system of the VO porphyrin and the linear combination of empty V *d*_*xz,yz*_ orbitals (Figures 3g and S8). Notably, the extent of overlap with these orbitals increases as *θ* decreases, and *J* increases by ca. a factor of 3 for θ = 60°. Since the PES relative to θ is pretty shallow, the $J_{VO_{x}FP}$ value can vary over a range of ∼1 cm^–1^, in agreement with the value previously used to fit the TREPR spectrum of **VO-FP**.^56^

Harnessing the information provided by DFT calculations, we proceeded to simulate the TREPR spectra by using the following spin Hamiltonian:

$$Ĥ=J{Ŝ}_{FP}\cdot \left({ŝ}_{1}+{ŝ}_{2}\right)+d\overset{2}{\underset{i=1}{\sum }}\left({Ŝ}_{FP,x}{ŝ}_{i,x}+{Ŝ}_{FP,z}{ŝ}_{i,z}-2{Ŝ}_{FP,y}{ŝ}_{i,y}\right)+D\left[{Ŝ}_{FP,z\prime }^{2}-\frac{S_{FP}\left(S_{FP}+1\right)}{3}\right]+E\left({Ŝ}_{FP,x\prime }^{2}-{Ŝ}_{FP,y\prime }^{2}\right)+\mu_{B}B\cdot \left[g_{FP}\cdot {Ŝ}_{FP}+\overset{2}{\underset{i=1}{\sum }}g_{VO}\cdot {ŝ}_{i}\right]+\overset{2}{\underset{i=1}{\sum }}{Î}_{i}\cdot A_{VO}\cdot {ŝ}_{i}$$ 4

where *S*_*FP*_ = 1 is the spin of the free-base porphyrin (after ISC) and $s_{1},s_{2}=1/2$ are the spins of the VO qubit, respectively. The first two terms model the exchange (*J*) and dipole–dipole (*d*) interactions between the FP and the two VO. The next two terms describe the zero-field splitting of the FP triplet (parametrized by *D* and *E*). The following are the Zeeman interactions with the external field of FP (isotropic, characterized by spectroscopic factor *g_FP_*) and VO (with spectroscopic tensor ***g_VO_***). The last term is the hyperfine interaction (with hyperfine tensor ***A**_**VO**_*) of each ^51^VO spin with its *I*_*i*_ = 7/2 nuclear spin. The terms in the spin Hamiltonian are defined with respect to the local principal axes *xyz* (for VO) and *x’y’z’* (for FP). The longitudinal component of the dipole–dipole interaction lies along *y*, while x′ and *y′* are rotated by 45° with respect to *y*. The angle between the *z* and *z*’ (orthogonal to the respective porphyrin planes) was adjusted to 80°—within the computed θ range from the shallow PES—to optimize the simulations. The exchange coupling was set to *J* = −0.7 cm^–1^, as obtained from the DFT calculations. All spin Hamiltonian tensors for the individual spins are well established in the literature,^24,26,56^ and were kept constant in the simulations (see Table S6 for the parameters). The dipolar interaction was computed from the DFT-optimized geometry, and BS-DFT calculations place the system in the strong-exchange regime, where the precise magnitude of *J* has only a minor influence on the spectroscopic features of the resulting spin levels. Consequently, the only fitting parameters were the out-of-equilibrium populations of the eigenstates.

The simulations in Figure 3a,b show that the spectra for both orientations are well reproduced by populating only the quintet multiplet. In contrast, populating the higher excited triplets produces two distinct peaks in either the parallel or perpendicular orientation, inconsistent with the experimentally observed single broad emissive signal. This is also evident from the corresponding energy-level diagrams and EPR transitions for the two representative orientations (*y* || *B*_0_ and *x* || *B*_0_) shown in Figure 3d,e (*z* || *B*_0_ is reported in Figure S17). The spectra are accurately reproduced by assuming out-of-equilibrium populations of [0, 0.09, 0.25, 0.33, 0.33], taken as independent of the magnetic field orientation for simplicity.^106^ Importantly, Figure 3d,e show that both the resonance position and line width can only be reproduced when the largest population differences occur between the central transitions of the quintet state. Although we cannot exclude that minor changes in the populations of the quintet states contributing to the wing transitions could be partially compensated by adding population to the T_2_ triplet, the overall population distribution is constrained.

### Spin Polarization Transients at Different Temperatures

The potential of molecular qubit technologies to operate above liquid nitrogen temperature motivated us to investigate the spin properties of the photoexcited quintet state and its non-Boltzmann spin populations across a wide temperature range, up to room temperature. The temperature-dependent TREPR survey is shown in Figure S14. Figure 4 presents the 2D TREPR contour plot at room temperature along with spectra at two representative time delays after photoexcitation. The transient signals for the seven studied temperatures are shown in Figure 4c.

**Figure 4:**
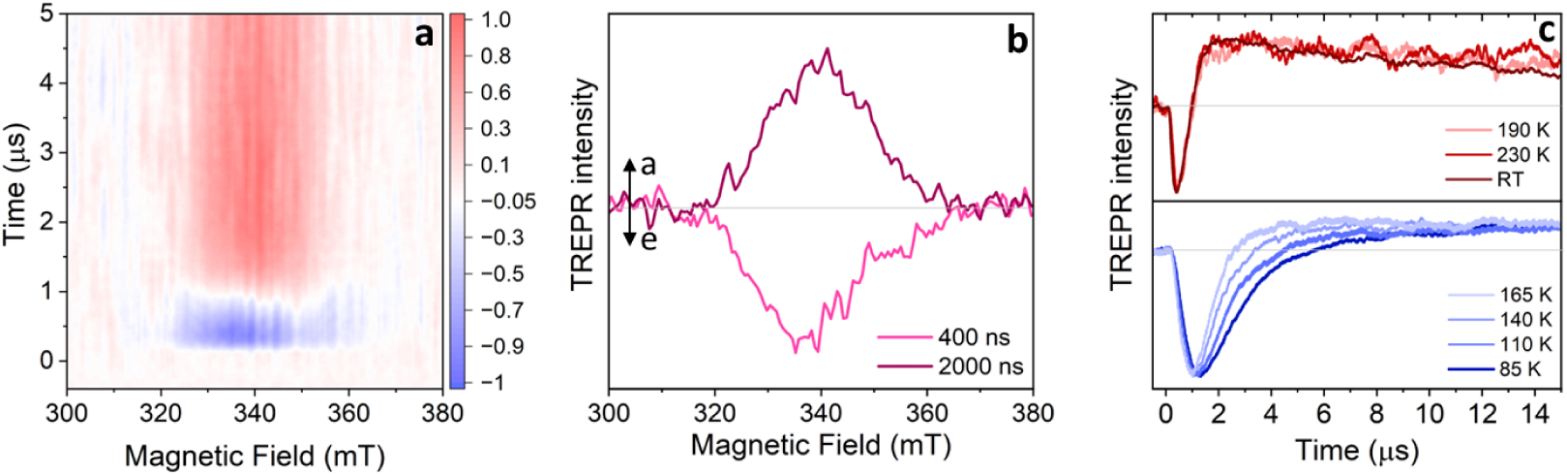
(a) Normalized 2D experimental TREPR contour plot of **VO-FP-VO** in toluene acquired at RT after a 550 nm laser pulse (7 ns, 2 mJ). Color legend: red = enhanced absorption, blue = emission, white = baseline. (b) Normalized 1D experimental TREPR spectra taken at representative times after the laser pulse (integrated time window = 200 ns). Arrows legend: a = enhanced absorption, e = emission. (c) Normalized TREPR transients taken at the field corresponding to the minimum of the spectra shown in Figure S14, using a 10 mT integration window. Measurements were performed at various temperatures between 85 K and room temperature. Transients acquired below the freezing point of toluene are shown in blue shades, while those recorded above the freezing point are shown in red shades.

At all temperatures, the time evolution of the spin polarization follows a similar trend: a net emissive signal is observed at short delays, which evolves into an absorptive polarization over a few microseconds. The shape and width of the TREPR signal do not change, suggesting that there is no transfer of population to the two thermally accessible triplet states. Above the freezing point, the transients do not depend on temperature, and the emissive signal persists for ∼1 μs, suggesting that molecular tumbling dominates the spin–lattice relaxation in this regime. Below the freezing point, the rate of transition from emissive to absorptive decreases with decreasing temperature. This dynamics is primarily driven by spin–lattice relaxation.^107^ The complete time evolution at the lowest investigated temperature (85 K) is shown in Figure S15. While the transition to absorptive signal occurs in ca. 6 μs, the overall spin polarization decays to zero only after ∼100 μs. The persistence of spin polarization well beyond the expected spin–lattice relaxation of the excited state suggests that the decay of the quintet state is spin-selective, producing an absorptive non-Boltzmann population in the excited state manifold.^102,105^ At all temperatures, no ground-state spin polarization is detected. This is attributed to the relatively slow decay of the ^3*^FP excited state compared to the spin–lattice relaxation time of vanadyl spins in the ground state,^24,26^ which prevents the accumulation of a net population difference.^108–110^

### Proposal for Light-Driven Entangling Gates

We now explore the potential of employing a similar molecular architecture for a light-driven quantum gate and outline the further steps in engineering the molecular structure necessary for its implementation. In this scheme, the two VO units serve as spin qubits, while the central chromophore acts as a controllable mediator of their mutual interaction, thereby enabling the implementation of light-driven two-qubit gates. In the FP ground state (*S*_*FP*_ = 0), the two qubits are effectively decoupled,^111^ allowing single-qubit rotations to be performed—in the presence of a static external magnetic field—via microwave pulses resonant with their respective energy gaps. Conversely, upon photoexcitation the FP triplet state (*S*_*FP*_ = 1) can mediate an interaction between the VO qubits (see below).

To this end, the VO-FP exchange interaction *J* must be sufficiently small to preserve the coherence of the two qubits during gate operations. In particular, *J* should be significantly smaller than the difference in EPR frequencies between VO and FP in the absence of interaction. This quantity—which includes the difference in the g-factors along the external field as well as the zero-field splitting for the FP and the hyperfine coupling for the VO—is on the order of 0.1–0.2 cm^–1^ for a magnetic field in Q- or W-band. Accordingly, the target exchange interaction should be in the 10^–2^ cm^–1^ range. In this regime, the state of the two qubits remains essentially factorized from that of the switch also after the photoexcitation. Hence, if the switch is prepared by photoexcitation in a pure state (e.g., *m*_*FP*_ = −1) we can define the computational basis as the four product states of the two VO qubits $\left|00\right\rangle \equiv \left|↑,m_{FP}=-1,↑\right\rangle$ , $\left|01\right\rangle \equiv \left|↑,m_{FP}=-1,↓\right\rangle$ , $\left|10\right\rangle \equiv \left|↓,m_{FP}=-1,↑\right\rangle$ , $\left|11\right\rangle \equiv \left|↓,m_{FP}=-1,↓\right\rangle$ . As detailed in the SI, the transverse component of the qubit-switch exchange interaction induces a mixing between |01⟩/|10⟩ states of the computational basis and excited states with *m_FP_* = 0 . In the perturbative limit we are considering, this mixing is small and cannot induce transitions to these excited states, but it introduces corrections to the low-energy Hamiltonian of the two qubits. In practice, one can derive by second-order perturbation theory that the dynamics of the four computational basis states is ruled by an effective Hamiltonian of the form

$${Ĥ}_{qq}=\Gamma \left({ŝ}_{x1}{ŝ}_{x2}+{ŝ}_{y1}{ŝ}_{y2}\right)+\lambda \left({ŝ}_{z1}+{ŝ}_{z2}\right)$$ 5

with $\Gamma \approx \frac{J^{2}}{\left(g_{VO}-g_{FP}\right)\mu_{B}B}$ , $\lambda \approx g_{VO}\mu_{B}B-J+\frac{J^{2}}{\left(g_{VO}-g_{FP}\right)\mu_{B}B}$ (where we have neglected, without loss of generality, hyperfine couplings and zero-field splitting on the FP; see Supporting Information for a detailed derivation). This effective Γ­(ŝ_*x1*_ŝ_*x2*_+ŝ_*y1*_ŝ_*y2*_) interaction yields a coupled free evolution of the two VO spins, which can be exploited to implement a two-qubit gate. It should be noted that the FP switch is not manipulated by microwave pulses; rather, it is activated by the laser pulse and passively mediates the effective qubit–qubit coupling. Due to Ĥ_*qq*_, logical states |00⟩ ≡ |↑↑⟩ and |11⟩ ≡ |↓↓⟩ do not evolve (in interaction picture), while |01⟩ ≡ |↑↓⟩ and |10⟩ ≡ |↓↑⟩ are transformed as follows

6 $$\left|01\right\rangle \to \cos\frac{\Gamma t}{2}\left|01\right\rangle +i\sin\frac{\Gamma t}{2}\left|10\right\rangle$$

7 $$\left|10\right\rangle \to i\sin\frac{\Gamma t}{2}\left|01\right\rangle +\cos\frac{\Gamma t}{2}\left|10\right\rangle$$

In particular, by choosing the duration of the gate as $t=\frac{\pi }{2\Gamma }$ we obtain the $\sqrt{i\mathrm{SWAP}}$ gate, which generates the maximally entangled state (|01⟩ + *i*|10⟩)/√2 starting, e.g., from a factorized |01⟩ state. Note that the same result could be obtained by computing the time evolution due to the full three-spin Hamiltonian and then expanding eigenvectors and eigenvalues in power series to lowest order in *J*/(*g_VO_– g_FP_*)*μ*_*B*_B.

The above derivation assumes the switch was initialized in a pure *m*_FP_ = −1 state. An effective coupling of the same form is also obtained for an initial *m*_FP_ = 1 state of the FP switch, but in general, with a different Γ and hence with a different duration of the $\sqrt{iSWAP}$ gate. In the free-base porphyrin case,^56^ the FP after photoexcitation is in a mixed state with equal probability of being in *m*_*FP*_ = −1 and *m*_*FP*_ = 1. This will lead, in general, to a different evolution in the two subspaces, resulting in an inefficient implementation of the gate. However, the two dynamics become very similar working at high field, e.g., ∼3.4 T at W-band.^112^ The simulation of the time evolution of the population of |01⟩ state for the two cases (FP either in *m*_*FP*_ = −1 or *m_FP_* = 1) is reported in Figure 5a, along with the mixed case. The time at which the curve intersects the horizontal line corresponds to the duration of the $\sqrt{iSWAP}$ , i.e., about 10 ns with known parameters for the individual subunits and *J* = 0.01 cm^–1^.

**Figure 5:**
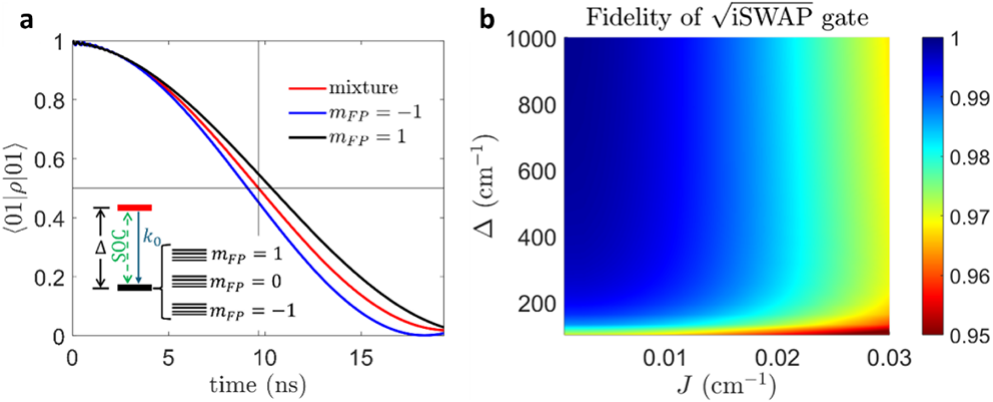
(a) Time evolution of the diagonal elements of the density matrix within the computational subspace for different initial states of the FP switch (blue vs black curves) or for an incoherent mixture of the two (red curve). Inset: minimal model including both orbital and spin degrees of freedom, used to assess the performance of the proposed scheme. Two orbital states with a large gap Δ are considered. Each group of levels (thick black and red lines) includes 12 spin states, i.e., the four possible product states of the two qubits for each state of the spin *S = 1* of the FP switch. (b) Fidelity of the $\sqrt{iSWAP}$ gate as a function of the exchange interaction (*J*) between FP and VO qubits and of the gap Δ between the orbital states. We assumed an external magnetic field of 3.3 T along the *z* axis (for the VO qubits) and we considered the qubit transition with *m_I_* =–7/2. Spin Hamiltonian parameters are the same used for EPR simulations, and we assumed a spin–orbit coupling of 6 cm^–1^ and an internal conversion rate between different orbital states *k*_0_ = 10^3^ ns^–1^. In a, *J* = 0.01 cm^–1^ and Δ = 300 cm^–1^.

We now analyze the performance of the proposed two-qubit gate by computing its fidelity F = ⟨*ψ*_*T*_|*ρ*|*ψ*_T_⟩, i.e., the squared absolute value of the scalar product between the target state |*ψ*_*T*_⟩ and the actual density matrix *ρ* obtained from numerical simulations of the system time evolution in the presence of imperfections. *F* is therefore a measure of the precision in the implementation of the gate.

In particular, we consider two main factors that may influence the gate. The first one is the VO-FP exchange interaction, which must be limited to maintain a good definition of the computational basis, with the states of the two qubits factorized from that of the switch. The second one is spin–orbit coupling on the triplet state of the FP, which induces a mixing between different orbital states, ultimately yielding decoherence within the computational subspace and hence lowering the gate fidelity. We describe this effect by following the minimal model proposed proposed in for VO porphyrins in ref^105^ for VO porphyrins, whose level diagram is sketched in the inset of Figure 5a. We consider (besides the spin degrees of freedom) two possible orbital states for the ^3*^FP with a gap Δ (thick black and red lines). Excited orbital states (red) are coherently mixed with the computational subspace (black) by spin–orbit coupling (green dashed arrow) and a fast (spin-conserving) internal conversion rate *k*_0_ between different orbital states on the ^3*^FP is included.

Clearly, the larger the mixing induced by spin–orbit coupling, the worse the effect on the gate fidelity. This mixing scales as 1/Δ and this prompted us to investigate the gate performance as a function of Δ to provide hints for the synthesis of suitable molecules for implementing the proposed scheme. Results of the simulated dynamics are shown in Figure 5b, where we report the fidelity in the implementation of the $\sqrt{iSWAP}$ as a function of *J* and Δ, with the internal conversion rate set to 10^3^ ns^–1^. We note that fidelities above 0.98 can be obtained over a relatively wide range of parameters. The fidelity decreases with *J*, because of too large mixing between the spins of the qubits and of the switch, and increases with Δ, since the effect of spin–orbit coupling on the computational manifold is reduced. Remarkably, we note a saturation above ∼ 300 cm^–1^, where the low-energy states used for quantum computation are practically isolated and hence further increase of Δseems less relevant.

## Conclusions and Perspectives

In this study, we synthesized a new porphyrin trimer comprising two VO qubits connected in the meso–meso positions by a free-base porphyrin chromophore. In the ground state, the two VO qubits are essentially independent, whereas upon photoexcitation, the FP excited singlet state rapidly undergoes exchange-mediated EISC to form the FP excited triplet state in less than 1 ps. Orientation-dependent TREPR, supported by DFT calculations and spectral simulations, reveals the formation of a long-lived photoexcited quintet state (*S = 2*) arising from ferromagnetic coupling between the FP triplet and the two VO doublets, in agreement with theoretical models for strongly and moderately coupled VO-FP systems.^113^ Temperature-dependent spin-polarization studies (85 K to room temperature) show that the non-Boltzmann populations evolve from an initial emissive signal to a fully absorptive signal, persisting for several microseconds even at room temperature.

The combination of a switchable exchange interaction with long-lived spin polarization at ambient conditions motivated us to explore the potential of this system for quantum information science. Theoretical analysis identified key requirements for the practical realization of a light-activated $\sqrt{iSWAP}$ gate. Importantly, implementing this entangling gate through our light-induced approach does not require coherent detection of the photoexcited state, i.e., when the light-driven magnetic exchange between the vanadyl qubits is active. Conversely, when the magnetic exchange is switched off, our measurements reveal a clear spin echo at 85 K, with a phase memory time comparable to that of a vanadyl monomer (Figure S11). For an efficient light-activated √iSWAP gate, three conditions must be satisfied: (i) a weak VO-FP exchange coupling, (ii) a large energy gap (Δ) between the lowest FP triplet and its higher excited states, and (iii) a means to rapidly return the ^3*^FP to its *S* = 0 ground state, thereby switching off the qubit–qubit interaction on demand. The requirement to reduce the magnitude of *J* is not critical, as the meso–meso link can be extended as needed, most likely after in silico optimization of the design. Accessing lower exchange-coupling regimes is also expected to further extend the excited-state lifetime and enhance both *T*_1_ and *T*_2_ relaxation times, thereby offering greater potential for implementing gate operations at higher temperatures. The use of linkers with reduced torsional degrees of freedom is also necessary to avoid conformational distributions of *J* values. The second requirement demands a gap of at least 300 cm^–1^ between the low-energy triplet subspace and excited orbital states of ^3*^FP, in order to suppress spin–orbit-mediated mixing that could otherwise accelerate relaxation and induce decoherence within the computational subspace. Previous calculations show that this condition is indeed fulfilled in free-base porphyrins, where this gap is in the order of 0.26 eV (> 2000 cm^–1^).^114^ The more challenging aspect is the ability to quickly suppress the qubit–qubit interaction to operate the $\sqrt{iSWAP}$ gate. Quenching the triplet state of the porphyrin via stimulated emission of the ^3*^FP does not appear promising, since this process is efficient only when the spin multiplicity of the excited and ground states is the same. However, triplet quenching can be activated by other mechanisms, such as energy transfer or electron transfer from a nearby second chromophore.

All in all, the ability to vary the metal centers, chromophores, and molecular bridges offers an almost unlimited design space for developing quantum gates tailored to specific needs. It is important to acknowledge that molecular platforms are still in an early stage compared with more mature top-down technologies, e.g., superconducting transmons, where qubit addressability and gate operations have been demonstrated in complex systems comprising several interconnected qubits. Nevertheless, although further molecular engineering will be required, the light-activated VO-FP-VO architecture, in our view, represents a meaningful first step toward the realization of individually addressable qubits with switchable interactions—an essential prerequisite for scalable quantum architectures.

## Supplementary Material



## Notes

Materials and methods, DFT calculations, W-band EPR, Transient absorption, Time-resolved EPR (PDF)
