# Supplementary material for: Light-Activated Qubit Coupling in a Vanadyl Porphyrin Trimer
Source: J Am Chem Soc. 2026 Mar 6;148(10):10408–20. doi: 10.1021/jacs.5c17205 (PMC13003507; doi:10.1021/jacs.5c17205)
Supplement: Supplementary file 1 [file ja5c17205_si_001.pdf]

Supplementary Information for

Light-Activated Qubit Coupling in a Vanadyl Porphyrin Trimer

Alberto Privitera,[1,2*] Alessandro Chiesa,[3] Fabio Santanni,[4,5] Davide Ranieri,[4,5] Prem P. Sahu,[4] Matthew D. Krzyaniak,[2] Andrea Caneschi,[1] Ryan M. Young,[2] Mathias O. Senge,[5] Federico Totti,[4] Michael R. Wasielewski,[2*] Stefano Carretta,[3*] Roberta Sessoli[4*]

[1] *Department of Industrial Engineering, University of Florence & UdR INSTM Firenze, 50121, Firenze, Italy*

[2] *Department of Chemistry, Center for Molecular Quantum Transduction, and Institute for Quantum Information Research and Engineering, Northwestern University, 60208-3113, Evanston, IL (USA)*

[3] *Department of Mathematical, Physical and Computer Sciences, University of Parma & UdR INSTM, 43124, Parma, Italy.*

[4] *Department of Chemistry "U. Schiff", University of Florence & UdR INSTM Firenze, 50019, Sesto Fiorentino, Italy*

[5] *School of Chemistry, Trinity Biomedical Sciences Institute, Trinity College Dublin, D02R590 Dublin, Ireland*

Table of Contents

1. Materials and methods 4

2. DFT calculations 9

3. W-band Echo-detected EPR 34

4. Transient absorption spectroscopy 35

5. Time-resolved electron paramagnetic resonance 36

6. Derivation of the Hamiltonian for the $\sqrt{iSWAP}$ gate 43

7. References 46

1. Materials and methods

General remarks. Moisture and air sensitive materials and reactions were handled under a N_2 atmosphere using standard Schlenk techniques. All glassware was dried by heating under vacuum prior to use. Where not specified, chemicals were purchased and used as such without further purification. Anhydrous toluene was obtained by distillation from K. Commercially available anhydrous DMF (99.8%, Extra Dry, Acroseal, Thermo Scientific) and CH_2Cl_2 (99.8%, Extra Dry, Acroseal, Thermo Scientific) were employed as such. $Pd(PPh_3)_4$ (99.9%, Strem) and Cs_2CO_3 were pumped in vacuum for 1 h before use. 5,15-Diphenylporphyrin (H_2DPP) was obtained using reported procedure,[1-2] and its vanadyl complexes [VO(DPP)] and [VO(DPPI)] were obtained using the procedures reported in refs.[3-4] 5,15-Diphenyl-10,20-bis(4,4,5,5-tetramethyl-1,3,2-dioxaborolan-2-yl)porphyrin (H_2DPPBpin$_2$) was obtained from H_2DPPBr$_2$ following a reported procedure.[5-6]

Synthesis of 5,15-bis[(10,20-diphenylporphyrin-5-yl-ato)oxovanadium(IV)]-10,20-diphenylporphyrin (VO-FP-VO). H_2DPPBPin$_2$ (5 mg, 0.007 mmol), [VO(DPPI)] (9 mg, 0.014 mmol, 2 eq.), and Cs_2CO_3 (18 mg 0.056 mmol, 4 eq.) were mixed together in a predried Schlenk flask and pumped in vacuum for 1 h. The solids were then dissolved in a 2:1 mixture of toluene/DMF (12 + 6 mL). The solution was purged with N_2 for 30 min, and then, $Pd(PPh_3)_4$ (0.1 eq.) was added. The reaction mixture was heated to 80 °C and stirred for 5 h (TLC check). At the end of reaction, H_2O (10 mL) was added and the organic phase collected. The obtained solution was further washed with 3 × 10 mL of H_2O. The organic phase was then collected, dried with Na_2SO_4, filtered, and the solvent removed in vacuum. The crude product was purified by column chromatography (flash SiO_2, hexane:CH_2Cl_2 1:2, R_f = 0.21) to yield

[$(VODPP)_2(H_2DPP)$] (2 mg, 0.001 mmol, 19%) as a red powder. UV-Vis (toluene): λ_{max} (ε / 10^4 L mol^{-1} cm^{-1}) = 414 nm (5.18), 476 nm (3.71), 522 nm (1.21), 549 nm (1.59), 598nm (0.49), 656 nm (0.11). FT-IR (ATR) = 657(w), 700(m), 723(m), 746(vw), 795(vs), 860(vw), 873(vw), 930(vw), 970(m), 1003(vs, stretching/*v* V=O), 1068 (m), 1155(w), 1178(w), 1211(vw), 1261(m), 1321(w), 1340(vw), 1377(w), 1400(vw), 1440(w), 1471(w), 1529(vw), 1556(vw), 1597(w), 1732(w), 2850(m), 2920(m), 2960(m), 3313(w, *v* N-H pyrrole) (Figure S2). M.P. > 350 °C. MALDI-ORBITRAP-HRMS (*m/z*): calculated for $C_{96}H_{59}N_{12}O_2V_2$ = 1514.3821; found = 1514.3810 ($M+H^+$) (Figure S3).

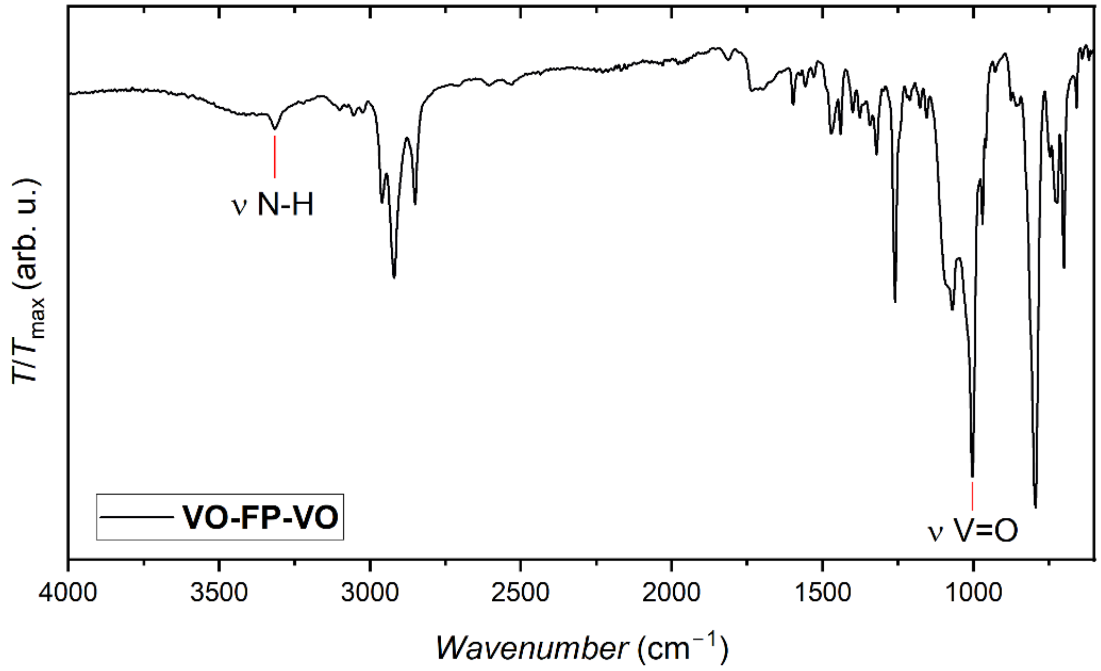

Figure S1. FT-IR spectrum of **VO-FP-VO**.

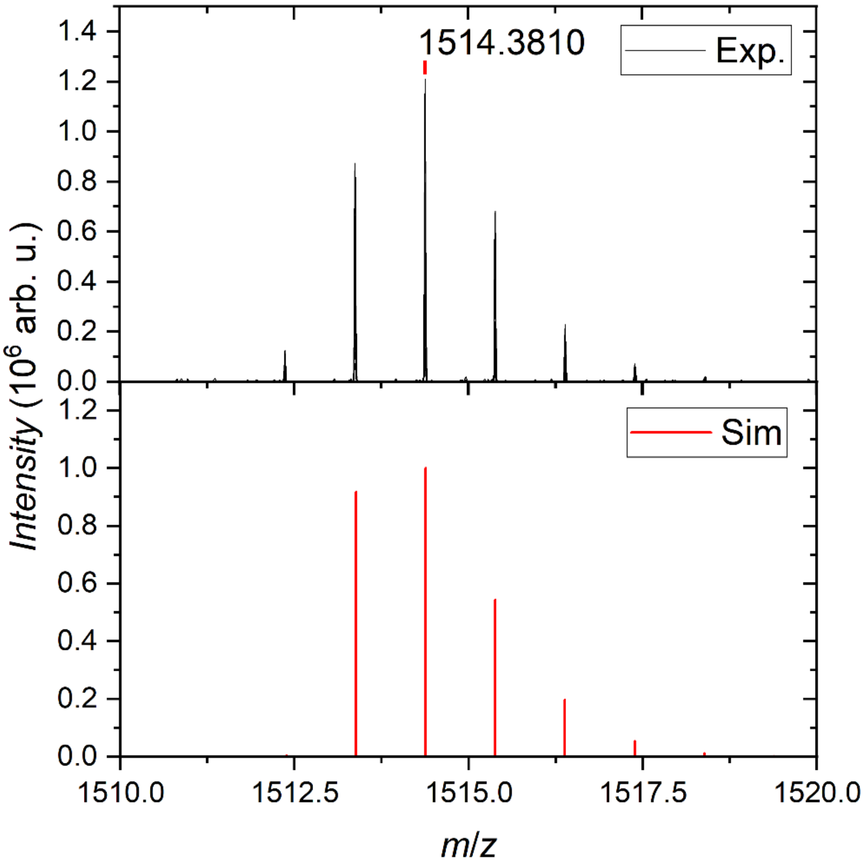

Figure S2. Comparison between experimental and simulated isotopic pattern distributions for MALDI-Orbitrap HRMS spectra of **VO-FP-VO**.

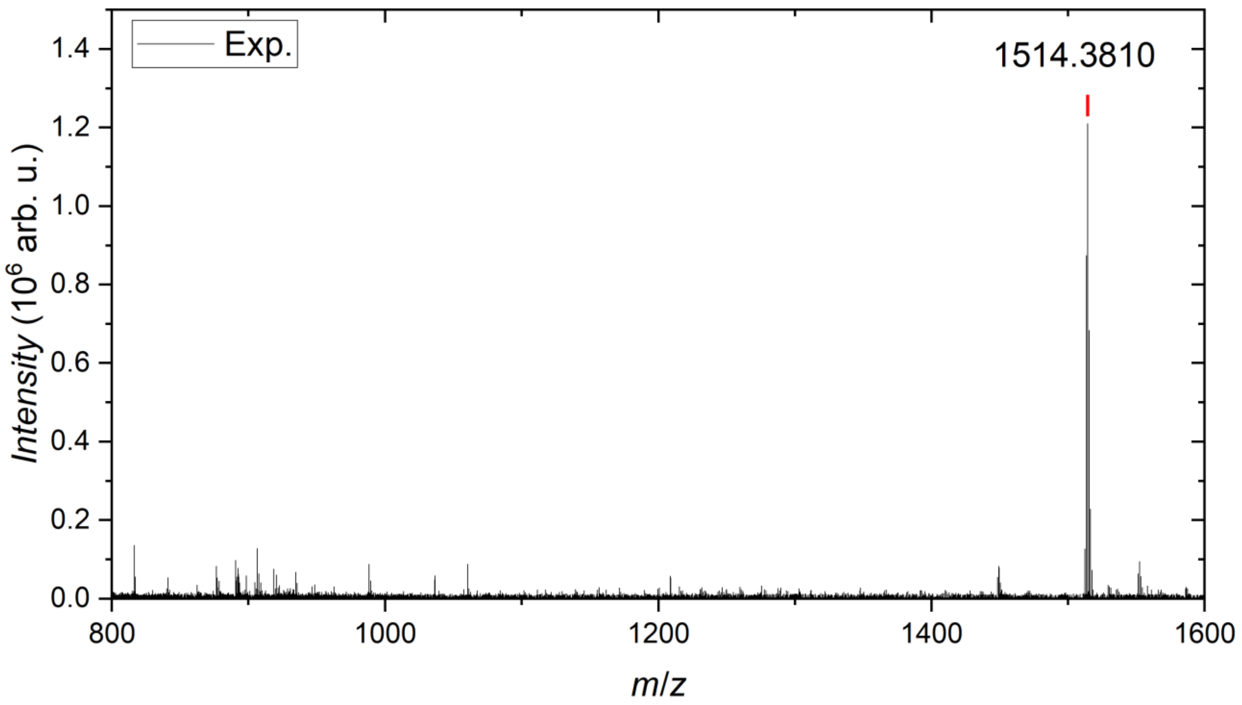

Figure S3. Experimental MALDI-Orbitrap HRMS spectrum of **VO-FP-VO**.

Instrumentation. UV-Vis measurements on samples were performed with a Shimadzu 1800 spectrophotometer. FT-IR measurements were performed in ATR mode using a Bruker Tensor 37 FTIR spectrometer. MALDI-Orbitrap HRMS samples were prepared as follows: 1 µL of sample stock solution was spotted on a stainless steel MALDI plate together with 1 µL of 2′,4′,6′-trihydroxyacetophenone monohydrate (THAP, from Merck-Sigma) solution (10 mg/mL in 50% ethanol) with dried droplet method. Instrumental analysis was performed on LTQ-Orbitrap XL (Thermo Scientific, San Josè, CA, USA) equipped with a vacuum MALDI source with a nitrogen laser emitting on wavelength equal to 337.1 nm at 60 Hz. The ion source worked with laser energy set at 12 µJ with automatic gain control method (22 average laser shots per spectra). The Orbitrap mass analyser worked in positive ion mode in the mass range from 700 to 2000 *m/z* with 100000 mass resolution.

2. DFT calculations

All quantum chemical calculations were performed using ORCA 6.0.0.[7] To investigate the preferred spatial configuration of the two vanadyl units in the **VO-FP-VO** trimer, two distinct configurations were independently modeled: one with the vanadyl oxo units aligned parallel on the same side of the chromophore linker, '*cis*', and another with the oxo groups oriented oppositely across the linker, '*trans*' (see Figure S4). Geometry optimizations for both configurations were conducted employing the B3LYP functional[8-9] in combination with the def2-SVP basis set. The electronic description of key atoms in the first coordination sphere (vanadium, nitrogen, oxygen, and carbon) was refined by upgrading their basis sets to the def2-TZVP level. Corresponding auxiliary basis sets for the RI approximation were generated via the AutoAux[10] procedure to ensure computational consistency. To achieve a balance between computational efficiency and accuracy, the RIJCOSX approximation was utilized to expedite the evaluation of exact exchange integrals, which is particularly advantageous for large molecular systems. Dispersion effects were incorporated through Grimme's D3BJ empirical correction[11-12] to adequately account for long-range van der Waals interactions. Numerical integration of the exchange-correlation terms was performed using the DEFGRID3 setting to ensure accurate and stable evaluation of DFT energies and gradients. Solvent effects were treated implicitly using the conductor-like polarizable continuum model (CPCM)[13] with toluene as the solvent medium. To verify that the optimized geometries correspond to true minima on the potential energy surface, harmonic frequency analyses were performed. The resulting Hessians exhibited exclusively real (positive) frequencies, confirming the absence of imaginary modes and the stability of both conformations. This comprehensive computational framework enabled the identification of the more stable conformation and facilitated an

assessment of how the differing spatial arrangements impact magnetic exchange interactions within the trimer system.

Ground-state magnetic exchange couplings were evaluated using the Broken Symmetry (BS) DFT approach. The isotropic exchange parameter J was calculated within the full projected formula:

$$J = \frac{[E(HS)-E(BS)]}{2S_1 \cdot S_2} \qquad \text{(eq. 1)}$$

where E(HS) and E(BS) represent the energies of the ferromagnetically (high-spin) and antiferromagnetically (broken symmetry) coupled states, respectively. Here, S_1 and S_2 denote the spin quantum numbers of the two V^{IV} ions, each with spin $\frac{1}{2}$.

To ensure accurate determination of both the sign and magnitude of J, SCF calculations were performed without convergence accelerators or auxiliary basis sets, employing the strict VeryTIGHTSCF convergence criterion (SCFCONV10), corresponding to an energy change threshold of 1.0×10^{-10} atomic units. Spin expectation values $\langle S^2 \rangle$ for both high-spin and broken symmetry states were monitored to confirm the reliability of the solutions (see Table S2). This protocol has been validated in previous studies when dealing with very small exchange coupling constants.[4, 14-15] To investigate how tilting affects magnetic exchange, we reduced the tilt angle for the two **VO-FP** units from the near-orthogonal DFT-optimized structure to a modelled one of 60° for each unit, and computed the corresponding J values using broken-symmetry DFT (see Figure S4).

For excited states, single-point TD-DFT calculations targeting triplet and quintet states were performed. The quintet state was treated as the high-spin reference, while multiple broken symmetry determinants were constructed by selectively flipping spins localized on the vanadyl centers, allowing exploration of different spin coupling scenarios (see Table S3). These

calculations primarily employed the B3LYP functional, with the CAM-B3LYP functional[16] used for comparison. The def2-TZVP basis sets were applied to V, O, N, and C atoms, while def2-SVP was used for H atoms. The same solvent and basis set treatment as in the ground state was applied.

Spin expectation values $\langle S^2 \rangle$ for both high-spin and all broken symmetry determinants were examined to verify the accuracy of the solutions (see Table S3). Additionally, spin density analyses were conducted to verify correct spin localization and flipping in the broken symmetry states (see Figure S8).

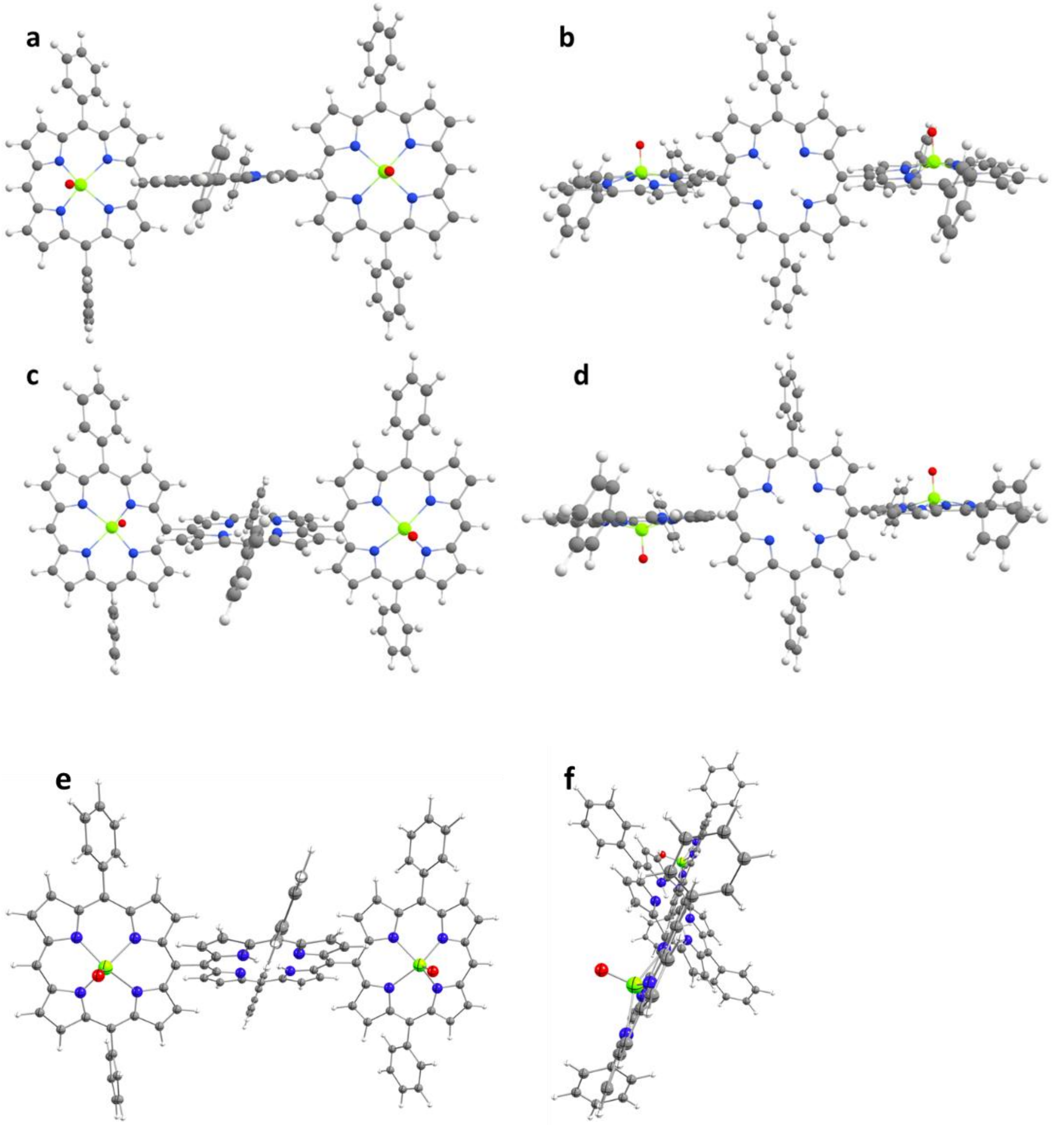

Figure S4. Orthogonal views of **VO-FP-VO** trimer conformations: '*cis'* (a,b) vs. '*trans'* (c,d) vanadyl oxo group alignments. The 60° FP-vanadyl tilt of the *cis* configuration is shown in top (e) and side (f) views. The code color is: V (green), N (blue), O (red), C (grey), and H (white).

Table S1. Key DFT-optimized structural parameters of the two vanadyl (V=O) units and their first coordination spheres in the **VO-FP-VO** systems, shown for both *cis* and *trans* configurations of the vanadyl units. Experimental data and previously reported DFT values for vanadyl dimers are included for comparison and validation of the computational results.[4]

<table>
<tr><th colspan="5">cis conformer</th><th colspan="3">trans conformer</th></tr>
<tr><th colspan="2"></th><th>DFT this study</th><th>DFT previous study</th><th>X-ray</th><th></th><th>DFT this study</th><th>DFT previous study</th></tr>
<tr><td rowspan="5">For V1</td><td>V1-O3</td><td>1.581Å</td><td>1.578Å</td><td>1.506Å</td><td>V1-O3</td><td>1.581Å</td><td>1.578Å</td></tr>
<tr><td>V1-N5</td><td>2.086Å</td><td>2.093Å</td><td>2.091Å</td><td>V1-N5</td><td>2.086Å</td><td>2.093Å</td></tr>
<tr><td>V1-N8</td><td>2.086Å</td><td>2.093Å</td><td>2.091Å</td><td>V1-N8</td><td>2.086Å</td><td>2.093Å</td></tr>
<tr><td>V1-N7</td><td>2.081Å</td><td>2.090Å</td><td>1.987Å</td><td>V1-N7</td><td>2.081Å</td><td>2.090Å</td></tr>
<tr><td>V1-N7</td><td>2.081Å</td><td>2.090Å</td><td>1.987Å</td><td>V1-N7</td><td>2.081Å</td><td>2.090Å</td></tr>
<tr><td rowspan="5">For V2</td><td>V2-O4</td><td>1.581Å</td><td>1.578Å</td><td>1.506Å</td><td>V2-O4</td><td>1.581Å</td><td>1.578Å</td></tr>
<tr><td>V2-N13</td><td>2.087Å</td><td>2.093Å</td><td>2.091Å</td><td>V2-N13</td><td>2.087Å</td><td>2.093Å</td></tr>
<tr><td>V2-N15</td><td>2.087Å</td><td>2.093Å</td><td>2.091Å</td><td>V2-N15</td><td>2.087Å</td><td>2.093Å</td></tr>
<tr><td>V2-N14</td><td>2.080Å</td><td>2.090Å</td><td>1.987Å</td><td>V2-N14</td><td>2.080Å</td><td>2.090Å</td></tr>
<tr><td>V2-N16</td><td>2.080Å</td><td>2.090Å</td><td>1.987Å</td><td>V2-N16</td><td>2.081Å</td><td>2.090Å</td></tr>
<tr><td></td><td>V1-V2</td><td colspan="3">16.63 Å</td><td>V1-V2</td><td colspan="2">16.70 Å</td></tr>
<tr><td rowspan="2">Tilting angles</td><td>θ1</td><td colspan="3">92.17°; 91.44°</td><td>θ1</td><td colspan="2">92.38°; 93.39°</td></tr>
<tr><td>θ2</td><td colspan="3">89.75°; 88.92°</td><td>θ2</td><td colspan="2">89.79°; 90.57°</td></tr>
</table>

Table S2. Total energies, ⟨S^2⟩ expectation values, and computed magnetic exchange coupling constants (J) for the *cis* **VO-FP-VO** trimer in *cis* and *trans* configurations, evaluated for (a) B3LYP with near-orthogonal geometry, and (b) B3LYP with a 60° tilt of each vanadyls with respect to the FP unit for *cis* configuration of the vanadyl units, respectively.

**(a) B3LYP with near-orthogonal geometry**					
	**HS (E_h)**	**⟨S^2⟩**	**BS (E_h)**	**⟨S^2⟩**	**J, cm^{-1}**
cis	-6387.622792562850	2.023	-6387.622792562790	1.023	**-1.32E-05**
trans	-6387.622613090530	2.023	-6387.622613090350	1.023	**-3.95E-05**
Energy difference between the two conformations = **-0.11 kcal/mol..**				J_{avg}	**-2.64E-05**
**(b) B3LYP with 60° tilt for parallel conformation**					
cis	-6383.440947511750	2.023	-6383.440947512740	1.023	**2.17E-04**

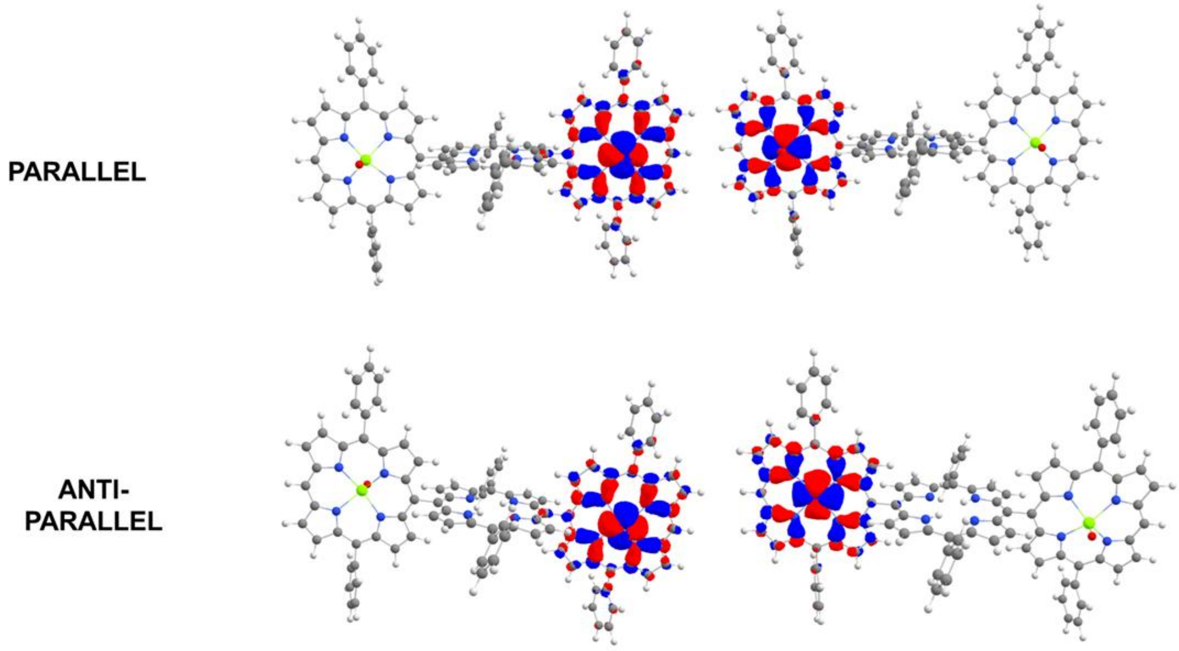

Figure S5. Ground-state broken-symmetry d_{xy} magnetic orbitals of the **VO-FP-VO** trimer at B3LYP with near-orthogonal geometry for '*cis'* (top) and '*trans'* (bottom) vanadyl configurations, highlighting magnetic localization. Positive and negative spin densities are represented by red and blue isosurfaces, respectively, rendered at a contour value of 0.015 $e^{-}/bohr^{3}$. Atom color scheme: V (green), N (blue), O (red), C (grey), and H (white).

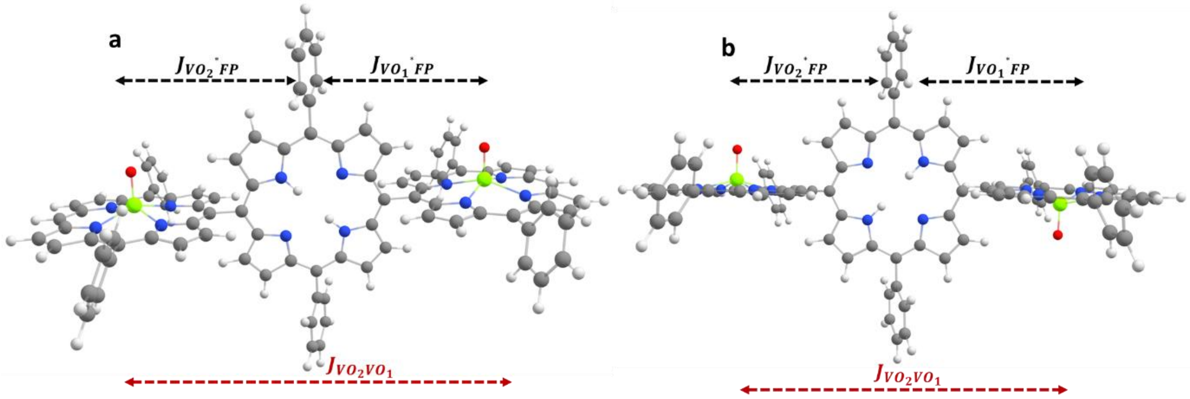

Figure S6. DFT-derived magnetic exchange coupling pathways in the excited state regimes of the **VO-FP-VO** trimer, illustrating the interactions $J_{VO_1VO_2}$, J_{VO_2FP}, and J_{VO_1FP} for *cis* (a) and *trans* (b) arrangements of vanadyl centers. Color coding of atoms: V (green), N (blue), O (red), C (grey), and H (white).

Table S3. Computed total energies (in E_h), spin expectation values ⟨S^2⟩, local spin densities (ρ) at the two vanadyl centers, and the magnetic exchange coupling constants (in cm^{-1}) for the **VO-FP-VO** trimer in the excited-state regime across different spin configurations: high-spin (HS) and three broken-symmetry (BS1, BS2, BS3) states. Results are presented for both '*cis*' and '*trans*' vanadyl alignments under three different conditions: (a) B3LYP with a near-orthogonal geometry, (b) CAM-B3LYP with a near-orthogonal geometry, and (c) B3LYP with a 60° vanadyl tilt w.r.t. the FP unit representing just a parallel conformation.

cis configuration					
**(a) B3LYP with a near-orthogonal geometry**					
**Spin Configurations**	**Energy (E_h)**	**⟨S^2⟩**	ρ^{V1}	ρ^{V2}	**J (cm^{-1})**
**HS (UUU)**	-6384.287302236470	6.166	1.1165	1.1163	J_{VO_1FP} = **-0.72**
**BS1 (DUU)**	-6384.287298874990	3.166	-1.1161	1.1163	J_{VO_2FP} = **-0.64**
**BS2 (UUD)**	-6384.287299417410	3.166	1.1165	-1.1162	$J_{VO_1VO_2}$ = **-**
**BS3 (DUD)**	-6384.287296058350	2.166	-1.1162	-1.1161	**5.31E-04**
**(b) CAM-B3LYP with a near-orthogonal geometry**					
**Spin Configurations**	**Energy (E_h)**	**⟨S^2⟩**	ρ^{V1}	ρ^{V2}	**J (cm^{-1})**
**HS (UUU)**	-6384.514939158290	6.166	1.1165	1.1163	J_{VO_1FP} = **-0.74**
**BS1 (DUU)**	-6384.514935804940	3.166	-1.1161	1.1163	J_{VO_2FP} = **-0.69**
**BS2 (UUD)**	-6384.514936033090	3.166	1.1165	-1.1162	$J_{VO_1VO_2}$ = **-**
**BS3 (DUD)**	-6384.514932680150	2.166	-1.1162	-1.1161	**9.00E-05**
**(c) B3LYP with** 60° *cis* configuration					
**Spin Configurations**	**Energy (E_h)**	**⟨S^2⟩**	ρ^{V1}	ρ^{V2}	**J (cm^{-1})**
**HS (UUU)**	-6384.256498185800	6.075	1.1081	1.1081	J_{VO_1FP} = **-2.35**
**BS1 (DUU)**	-6384.256487460590	3.075	-1.1080	1.1081	

**BS2 (UUD)**	-6384.256489514290	3.075	1.1081	-1.1081	**J_{VO_2FP} = -1.90**
**BS3 (DUD)**	-6384.256478789260	2.075	-1.1080	-1.1081	**$J_{VO_1VO_2}$ = -3.97E-05**
trans configuration					
**(a) B3LYP with a near-orthogonal geometry**					
**Spin Configurations**	**Energy (E_h)**	**⟨S^2⟩**	ρ ^{V1}	ρ ^{V2}	**J (cm^{-1})**
**HS (UUU)**	-6384.287336977160	6.075	1.1081	1.1081	**J_{VO_1FP} = -0.61**
**BS1 (DUU)**	-6384.287334203970	3.075	-1.1080	1.1081	**J_{VO_2FP} = -0.59**
**BS2 (UUD)**	-6384.287334293790	3.075	1.1081	-1.1081	**$J_{VO_1VO_2}$ = -7.00E-04**
**BS3 (DUD)**	-6384.287331523800	2.075	-1.1080	-1.1081	
**(b) CAM-B3LYP with a near-orthogonal geometry**					
**Spin Configurations**	**Energy (E_h)**	**⟨S^2⟩**	ρ ^{V1}	ρ ^{V2}	**J (cm^{-1})**
**HS (UUU)**	-6384.256498185800	6.075	1.1081	1.1081	**J_{VO_1FP} = -0.68**
**BS1 (DUU)**	-6384.256487460590	3.075	-1.1080	1.1081	**J_{VO_2FP} = -0.73**
**BS2 (UUD)**	-6384.256489514290	3.075	1.1081	-1.1081	**$J_{VO_1VO_2}$ = -8.00E-05**
**BS3 (DUD)**	-6384.256478789260	2.075	-1.1080	-1.1081	

**(a)** *cis* configuration	
**Near-orthogonal geometry**	60° **tilt**
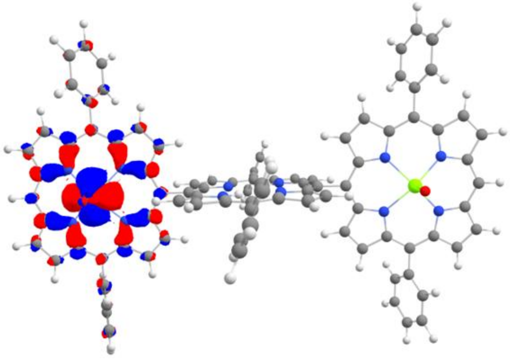	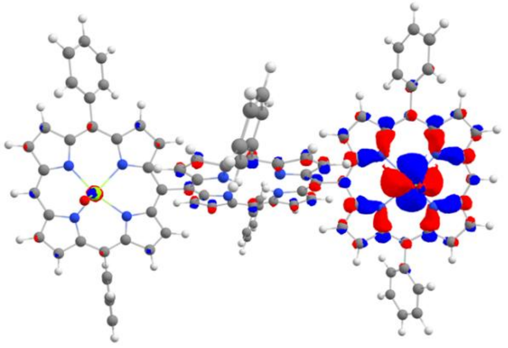
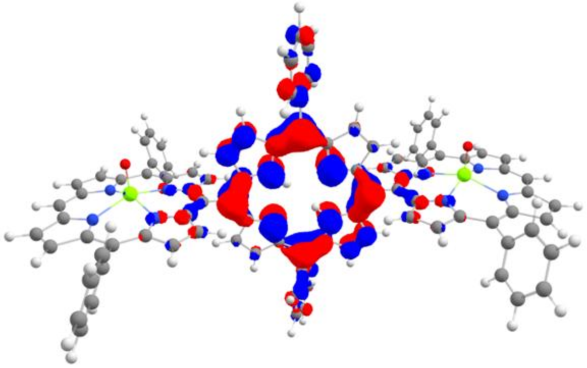	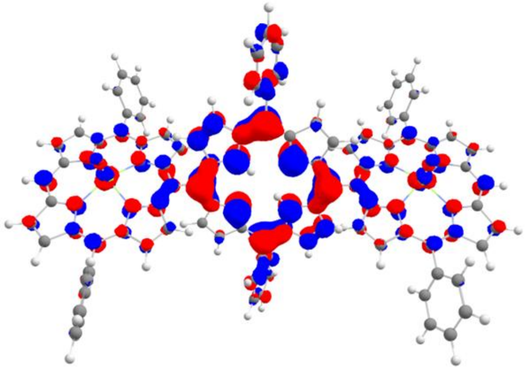
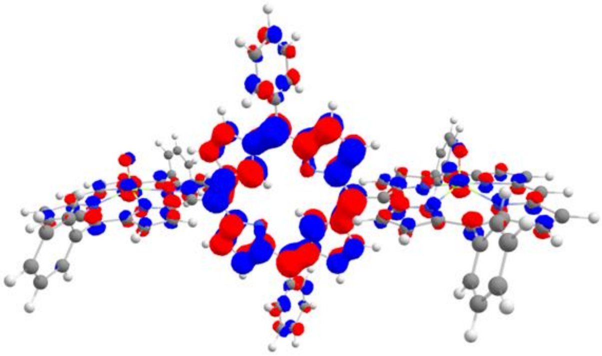	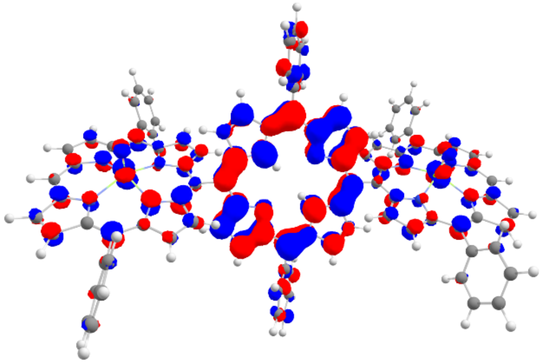
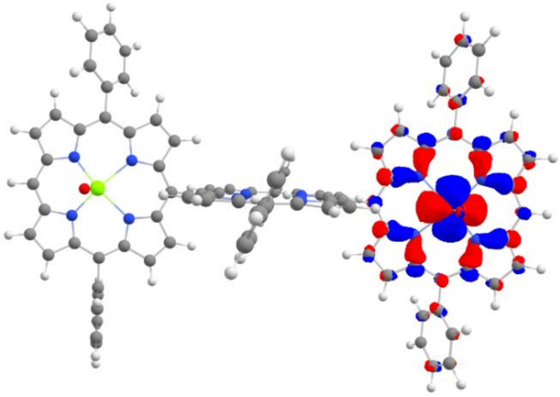	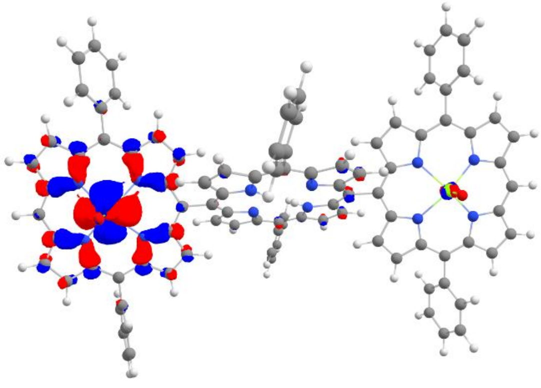

**(b)** *trans* configuration **(Near-orthogonal geometry)**
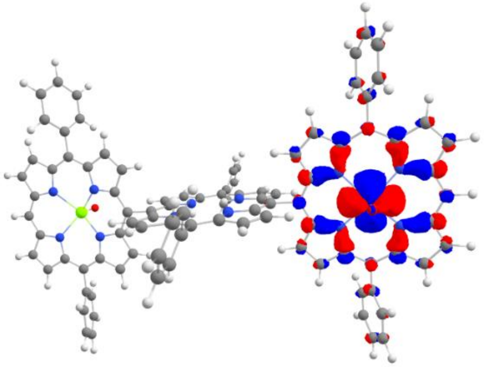
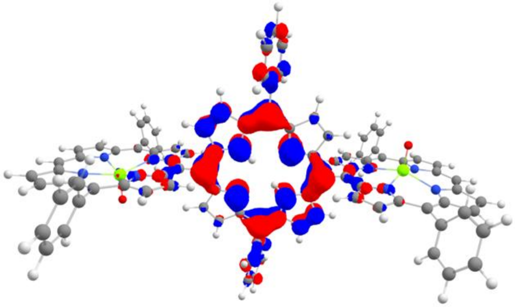
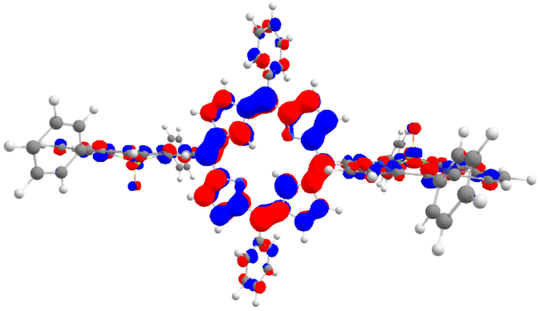
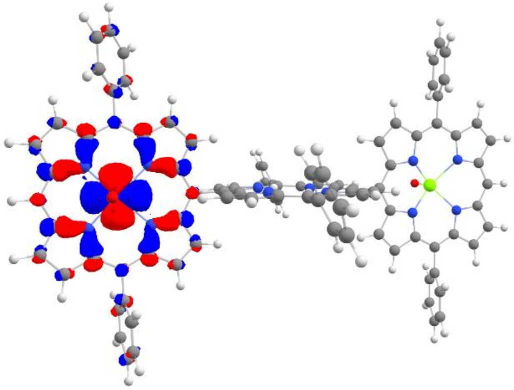

Figure S7. Excited state magnetic orbitals for the '*cis*' **VO-FP-VO** trimer at B3LYP, comparing (a) near-orthogonal geometry (left) and with a 60° tilt of the vanadyl units w.r.t FP unit, and (b)

antiparallel spin alignments for near orthogonal geometry. Red and blue iso-surfaces denote positive and negative spin densities at a contour level of 0.020833 e^-/$bohr^3$. Atom colors: V (green), N (blue), O (red), C (grey), H (white).

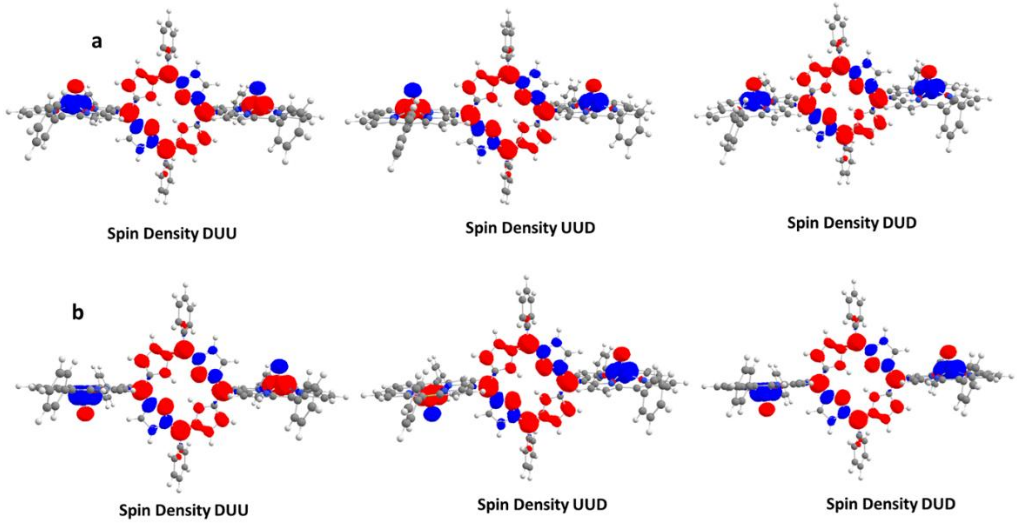

Figure S8. DFT-computed spin density iso-surfaces for the broken-symmetry determinants DUU, UUD, and DUD in the **VO-FP-VO** trimer complex, at B3LYP with near-orthogonal geometry, shown for '*cis'* (a) and '*trans'* (b) configurations of the vanadyl centers in the excited-state regime. Positive and negative spin densities are represented by red and blue isosurfaces, respectively, rendered at a contour value of 0.015 $e^{-}/bohr^{3}$. Atom color scheme: V (green), N (blue), O (red), C (grey), and H (white).

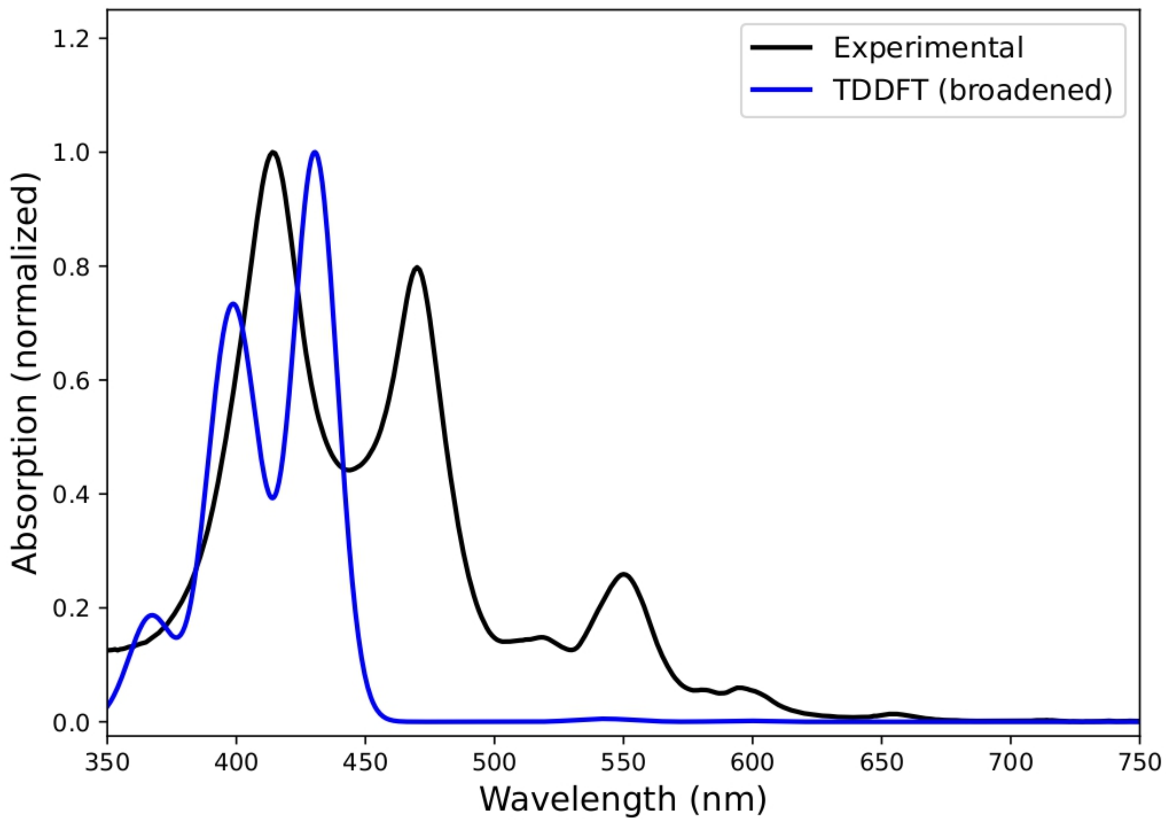

Figure S9. UV-Vis spectral comparison between experiment (toluene) and theory (TD-DFT with CAM-B3LYP) for the parallel conformation. The computed spectrum was broadened using a Gaussian function with FWHM = 20 nm to facilitate comparison with experimental data.

Table S4. Atomic positions of **VO-FP-VO** conformers: optimized (a) parallel and (b) antiparallel, and (c) modelled 60° tilt between each FP-vanadyl units, particularly for the cis configuration.

	(a) **parallel**		
V	0.000803000000	0.001465000000	0.000031000000
V	-11.597769000000	-11.880678000000	0.924958000000
O	-0.003345000000	0.010246000000	1.580552000000
N	2.016014000000	-0.013345000000	-0.538265000000
N	0.016285000000	-2.015990000000	-0.511285000000
N	-2.012641000000	0.001054000000	-0.527799000000
N	-0.021321000000	2.012195000000	-0.554776000000
O	-11.525270000000	-11.716303000000	2.495537000000
N	-11.583135000000	-13.940177000000	0.588735000000
N	-9.609695000000	-11.930432000000	0.316457000000
N	-13.636177000000	-11.934618000000	0.482085000000
N	-11.654665000000	-9.934075000000	0.193658000000
N	-6.844276000000	-7.065456000000	-1.573733000000
N	-4.809119000000	-4.865036000000	1.163301000000
N	-6.733906000000	-6.997443000000	1.377331000000
N	-4.916789000000	-4.934426000000	-1.786916000000
C	-6.552316000000	-5.109263000000	-5.911959000000
C	-6.046337000000	-6.180392000000	-5.167516000000
C	-5.592945000000	-7.318267000000	-5.843954000000
C	-5.644730000000	-7.384010000000	-7.233878000000
C	-6.152663000000	-6.312662000000	-7.966936000000
C	-6.606992000000	-5.175082000000	-7.302016000000
C	-5.987919000000	-6.110346000000	-3.679434000000
C	-6.806108000000	-6.978328000000	-2.934293000000
C	-7.782185000000	-8.015321000000	-1.297419000000
C	-8.362508000000	-8.549713000000	-2.520896000000
C	-7.758639000000	-7.899696000000	-3.541704000000
C	-4.848083000000	-4.951211000000	2.524035000000
C	-3.894768000000	-4.030324000000	3.131745000000
C	-3.288911000000	-3.381363000000	2.112006000000
C	-3.869898000000	-3.916428000000	0.887905000000
C	-8.177658000000	-8.441067000000	-0.021946000000
C	-7.696938000000	-7.957847000000	1.195507000000
C	-6.548157000000	-6.753109000000	2.715875000000
C	-7.449776000000	-7.615347000000	3.420399000000
C	-8.142136000000	-8.350089000000	2.498925000000
C	-5.666666000000	-5.818501000000	3.269476000000
C	-5.609748000000	-5.746530000000	4.757405000000
C	-6.059449000000	-4.605703000000	5.431265000000
C	-6.009169000000	-4.538159000000	6.821083000000
C	-5.506323000000	-5.610387000000	7.556308000000
C	-5.055610000000	-6.750766000000	6.893796000000
C	-5.108887000000	-6.818627000000	5.503851000000
C	-3.470962000000	-3.493180000000	-0.387749000000
C	-3.951217000000	-3.976123000000	-1.604918000000
C	-5.104927000000	-5.177391000000	-3.125584000000
C	-4.202648000000	-4.316269000000	-3.830182000000
C	-3.507395000000	-3.584536000000	-2.908035000000
C	-2.415156000000	-2.432155000000	-0.460808000000

C	-2.833509000000	-1.104410000000	-0.497900000000
C	-2.850613000000	1.095529000000	-0.564713000000
C	-4.222260000000	0.661026000000	-0.550788000000
C	-4.211146000000	-0.696601000000	-0.512121000000
C	-1.084678000000	-2.842981000000	-0.484760000000
C	-0.669019000000	-4.218329000000	-0.487315000000
C	0.688886000000	-4.221911000000	-0.514510000000
C	1.115740000000	-2.847922000000	-0.533709000000
C	2.450760000000	-2.441265000000	-0.562116000000
C	3.509112000000	-3.491430000000	-0.586530000000
C	3.706102000000	-4.278859000000	-1.725253000000
C	4.699188000000	-5.255072000000	-1.746404000000
C	5.505362000000	-5.457030000000	-0.627373000000
C	5.314687000000	-4.676845000000	0.511819000000
C	4.323546000000	-3.698590000000	0.531173000000
C	-2.451415000000	2.432600000000	-0.601337000000
C	-3.507457000000	3.484570000000	-0.642722000000
C	-3.727814000000	4.307327000000	0.466326000000
C	-4.711682000000	5.292467000000	0.431112000000
C	-5.484350000000	5.468784000000	-0.715493000000
C	-5.269222000000	4.654273000000	-1.826001000000
C	-4.287393000000	3.667204000000	-1.788976000000
C	2.854056000000	-1.104863000000	-0.584890000000
C	4.221555000000	-0.668519000000	-0.701098000000
C	4.204626000000	0.690938000000	-0.715824000000
C	2.830412000000	1.093097000000	-0.617935000000
C	2.400664000000	2.410107000000	-0.641327000000
C	1.081032000000	2.832316000000	-0.632366000000
C	-1.117227000000	2.843453000000	-0.617274000000
C	-0.687837000000	4.212448000000	-0.741380000000
C	0.671778000000	4.203305000000	-0.744934000000
C	-14.001576000000	-14.352578000000	0.705532000000
C	-12.678902000000	-14.765112000000	0.705737000000
C	-12.260512000000	-16.134609000000	0.799362000000
C	-10.902317000000	-16.139391000000	0.736269000000
C	-10.482978000000	-14.769008000000	0.594737000000
C	-9.155132000000	-14.355528000000	0.474206000000
C	-8.768947000000	-13.022708000000	0.321528000000
C	-7.409538000000	-12.593038000000	0.126741000000
C	-7.429088000000	-11.238624000000	0.025649000000
C	-8.801481000000	-10.829557000000	0.143334000000
C	-8.087279000000	-15.396236000000	0.494706000000
C	-7.929411000000	-16.276374000000	-0.580448000000
C	-6.928378000000	-17.244518000000	-0.560339000000
C	-6.074788000000	-17.345367000000	0.536997000000
C	-6.226228000000	-16.472336000000	1.612989000000
C	-7.225663000000	-15.502649000000	1.591004000000
C	-14.442733000000	-13.045640000000	0.573391000000
C	-15.823386000000	-12.664428000000	0.483377000000
C	-15.852101000000	-11.313465000000	0.331871000000
C	-14.485132000000	-10.860282000000	0.340223000000
C	-14.093299000000	-9.527601000000	0.198858000000
C	-12.761817000000	-9.113853000000	0.132722000000
C	-12.346770000000	-7.743019000000	-0.004982000000
C	-10.988937000000	-7.737178000000	-0.034780000000
C	-10.561059000000	-9.103362000000	0.086332000000

C	-15.160814000000	-8.491312000000	0.100016000000
C	-15.940370000000	-8.171957000000	1.216245000000
C	-16.939583000000	-7.205853000000	1.127472000000
C	-17.173341000000	-6.550141000000	-0.080125000000
C	-16.402139000000	-6.864421000000	-1.197920000000
C	-15.400899000000	-7.828274000000	-1.107640000000
C	-9.228049000000	-9.505568000000	0.073227000000
H	3.163850000000	3.177233000000	-0.706173000000
H	5.040728000000	1.370626000000	-0.803003000000
H	1.347328000000	5.042610000000	-0.833507000000
H	-1.347310000000	5.061409000000	-0.829808000000
H	-5.055490000000	-1.368070000000	-0.492799000000
H	-5.079293000000	1.315515000000	-0.565539000000
H	5.074975000000	-1.323756000000	-0.777198000000
H	1.348224000000	-5.075321000000	-0.518609000000
H	-4.876120000000	5.920270000000	1.300795000000
H	-1.335858000000	-5.066324000000	-0.468723000000
H	-5.864290000000	4.788428000000	-2.723489000000
H	-3.128299000000	4.166128000000	1.359298000000
H	-4.116098000000	3.035676000000	-2.654261000000
H	-6.250177000000	6.236992000000	-0.743578000000
H	3.081007000000	-4.117909000000	-2.597165000000
H	6.278238000000	-6.218504000000	-0.643097000000
H	4.171113000000	-3.093284000000	1.418377000000
H	5.935862000000	-4.831104000000	1.388099000000
H	4.844437000000	-5.856137000000	-2.638146000000
H	-8.905131000000	-9.089205000000	2.688179000000
H	-9.129613000000	-9.307458000000	-2.575453000000
H	-7.939653000000	-8.013774000000	-4.599236000000
H	-2.743410000000	-2.846446000000	-3.095538000000
H	-4.101151000000	-4.283210000000	-4.903003000000
H	-7.553407000000	-7.647438000000	4.492982000000
H	-3.713898000000	-3.916022000000	4.189183000000
H	-5.285156000000	-8.271543000000	-7.744391000000
H	-2.520709000000	-2.624548000000	2.168890000000
H	-7.008348000000	-4.338725000000	-7.865108000000
H	-5.192949000000	-8.149853000000	-5.273884000000
H	-6.912959000000	-4.226768000000	-5.394365000000
H	-6.194440000000	-6.364287000000	-9.050001000000
H	-6.455212000000	-3.773269000000	4.859529000000
H	-5.465739000000	-5.557284000000	8.639333000000
H	-4.751035000000	-7.703352000000	4.988180000000
H	-4.658378000000	-7.587873000000	7.458641000000
H	-6.365648000000	-3.648354000000	7.329743000000
H	-6.232455000000	-6.522390000000	0.638006000000
H	-5.418289000000	-5.408339000000	-1.046781000000
H	-12.929560000000	-16.978041000000	0.897612000000
H	-10.238285000000	-16.988285000000	0.778148000000
H	-6.556174000000	-13.249570000000	0.063763000000
H	-6.596258000000	-10.571305000000	-0.133428000000
H	-8.590696000000	-16.193567000000	-1.436440000000
H	-6.813470000000	-17.917777000000	-1.403616000000
H	-5.295254000000	-18.100007000000	0.553250000000
H	-5.567389000000	-16.546814000000	2.472001000000
H	-7.346848000000	-14.824497000000	2.428965000000
H	-16.656120000000	-13.353041000000	0.515145000000

H	-16.713891000000	-10.676037000000	0.211732000000
H	-13.013396000000	-6.897684000000	-0.068775000000
H	-10.329707000000	-6.888879000000	-0.134356000000
H	-15.754339000000	-8.680109000000	2.156421000000
H	-17.533353000000	-6.963584000000	2.002842000000
H	-17.952440000000	-5.798097000000	-0.149836000000
H	-16.581030000000	-6.360814000000	-2.142348000000
H	-14.802909000000	-8.076490000000	-1.978065000000
H	-14.759245000000	-15.123579000000	0.787047000000
	**(b) antiparallel**		
V	0.008530000644	-0.005124000389	-0.001445000130
V	-11.638290895147	-11.962870920085	-0.341911026310
O	0.048898003763	-0.037124002858	1.578301121393
N	-2.018736155271	-0.000914000077	-0.472977036354
N	0.017280001332	-2.008928154528	-0.564278043401
N	2.007911154439	0.002787000196	-0.595929045854
N	-0.036641002832	2.019051155278	-0.503389038702
O	-11.624682894063	-12.050395926832	-1.919968147672
N	-13.657254050418	-11.919932916803	0.183888014135
N	-11.654939896425	-9.923897763243	0.068295005244
N	-11.625031894079	-13.946855072687	0.304572023446
N	-9.630645740701	-11.941058918425	0.204574015723
N	-6.849682526795	-7.061852543149	-1.561760120111
N	-4.772742367100	-4.896920376620	1.171426090120
N	-6.697529515122	-7.029694540683	1.387497106695
N	-4.924465378760	-4.929698379131	-1.777820136731
C	-6.621785509290	-5.059502389121	-5.880628452306
C	-6.100952469236	-6.137180472033	-5.156243396551
C	-5.653187434774	-7.265453558812	-5.852100450085
C	-5.725008440317	-7.315360562616	-7.241775556990
C	-6.247507480531	-6.237607479730	-7.954905611847
C	-6.696436515019	-5.109552392969	-7.270403559159
C	-6.021625463148	-6.084245467961	-3.668387282123
C	-6.827901525132	-6.961863535461	-2.921991224745
C	-7.783659598639	-8.014967616437	-1.284133098749
C	-8.377580644327	-8.539979656805	-2.506317192770
C	-7.785985598825	-7.879423606010	-3.527065271252
C	-4.788880368315	-5.002146384730	2.531307194699
C	-3.823323294047	-4.091934314728	3.135649241166
C	-3.234005248716	-3.429255263761	2.114996162648
C	-3.837077295134	-3.945549303477	0.893541068708
C	-8.165604628057	-8.452179650083	-0.007919000633
C	-7.668128589795	-7.983267614023	1.207942092926
C	-6.491778499287	-6.801024523066	2.725943209668
C	-7.387957568229	-7.666273589641	3.433179264036
C	-8.097243622768	-8.386292645008	2.512547193242
C	-5.598253430570	-5.876770451970	3.277970252110
C	-5.518095424428	-5.824612448007	4.765647366512
C	-5.953367457872	-4.691446360838	5.461591420067
C	-5.880976452336	-4.642374357034	6.851234526936
C	-5.370161413048	-5.725741440396	7.564323581775
C	-4.933669379449	-6.858658527497	6.879808529160
C	-5.009087385266	-6.907966531284	5.490078422270
C	-3.460546266138	-3.503179269434	-0.382500029406
C	-3.959730304565	-3.970136305339	-1.598251122943

C	-5.132957394788	-5.155418396519	-3.116333239682
C	-4.245574326537	-4.281171329280	-3.823632294084
C	-3.538952272191	-3.558588273720	-2.902905223273
C	-2.410170185374	-2.436918187416	-0.457735035200
C	-2.834246218007	-1.110458085396	-0.450291034649
C	-2.861556220099	1.090379083874	-0.458738035270
C	-4.230680325412	0.649457049973	-0.420102032304
C	-4.213350324079	-0.708639054494	-0.417917032137
C	-1.079374083030	-2.841294218554	-0.527503040560
C	-0.658662050648	-4.214301324124	-0.574256044188
C	0.697977053695	-4.211101323893	-0.639149049174
C	1.118861086040	-2.835130218074	-0.637168049007
C	2.451005188526	-2.421862186296	-0.692785053289
C	3.512375270127	-3.466177266592	-0.772691059405
C	3.679754283016	-4.225388325005	-1.935077148804
C	4.676179359657	-5.195677399602	-2.008185154427
C	5.515440424216	-5.419894416863	-0.918093070628
C	5.354307411790	-4.668025359028	0.244467018812
C	4.359719335339	-3.695691284244	0.315807024302
C	-2.468579189837	2.429749186893	-0.470764036202
C	-3.529438271480	3.477535267450	-0.454294034949
C	-3.721761286243	4.269140328364	0.682325052464
C	-4.710851362315	5.249469403741	0.701724053971
C	-5.516864424318	5.452215419362	-0.417223032104
C	-5.329613409918	4.669122359128	-1.555046119575
C	-4.342478334000	3.686818283554	-1.572640120928
C	2.848388219050	-1.083469083335	-0.693649053367
C	4.210391323815	-0.638231049099	-0.837462064429
C	4.187947322115	0.721078055462	-0.817380062843
C	2.815574216557	1.114521085718	-0.670555051557
C	2.380427183087	2.429784186888	-0.648226049863
C	1.060033081519	2.845747218857	-0.590943045473
C	-1.136909087447	2.846817218959	-0.513067039486
C	-0.716258055073	4.220456324622	-0.613377047152
C	0.642723049448	4.217379324393	-0.655970050469
C	-14.048658080511	-14.337934102751	0.364289028018
C	-14.475762113359	-13.023676001677	0.264606020355
C	-15.853170219312	-12.620922970708	0.256360019721
C	-15.868960220498	-11.264057866351	0.170433013118
C	-14.496650114973	-10.828870832850	0.136248010482
C	-14.089883083697	-9.494955730271	0.075946005859
C	-12.753496980916	-9.091416699211	0.071174005498
C	-12.325193947941	-7.717934593612	0.093757007191
C	-10.966975843511	-7.722809593985	0.087414006739
C	-10.552972811628	-9.098708699789	0.071096005459
C	-15.140473164480	-8.437462648958	0.032380002482
C	-15.921616224544	-8.161185627710	1.158793089122
C	-16.898610299694	-7.169508551443	1.115356085770
C	-17.107587315773	-6.444428495667	-0.056618004381
C	-16.333564256219	-6.714514516436	-1.183915091035
C	-15.354520180950	-7.704017592544	-1.138818087599
C	-12.729705979061	-14.760512135241	0.409006031441
C	-12.324419947896	-16.124433240141	0.596623045888
C	-10.964784843321	-16.135572240995	0.608301046791
C	-10.531573810003	-14.775396136398	0.417644032135
C	-9.196302707288	-14.368409105078	0.383162029456

C	-8.794609676400	-13.035228002566	0.283890021856
C	-7.422179570832	-12.604385969424	0.249942019242
C	-7.430519571496	-11.249067865170	0.162017012440
C	-8.807222677355	-10.838795833624	0.136410010477
C	-8.142749626294	-15.419480185914	0.474627036523
C	-7.932623610089	-16.302244253817	-0.589483045337
C	-6.951597534666	-17.287390329625	-0.507509039033
C	-6.171370474653	-17.403765338557	0.641631049331
C	-6.376043490390	-16.529170271288	1.707535131338
C	-7.355074565679	-15.542218195394	1.623659124891
C	-9.222733709349	-9.511509731533	0.068550005283
H	3.138603241394	3.201692246266	-0.715059054999
H	5.018663385972	1.406487108165	-0.911035070060
H	1.312379100933	5.061674389308	-0.741845057047
H	-1.381075106218	5.068522389853	-0.660840050805
H	-5.054353388759	-1.384160106443	-0.394680030336
H	-5.090325391531	1.300199100002	-0.395211030389
H	5.063766389484	-1.287525099038	-0.954208073392
H	1.360225104611	-5.061191389283	-0.681995052452
H	-4.853271373273	5.852827450141	1.592347122486
H	-1.321446101653	-5.065520389598	-0.557642042904
H	-5.950660457687	4.824115371010	-2.431321187023
H	-3.096336238144	4.107209315905	1.553759119494
H	-4.193090322507	3.079729236870	-2.459204189119
H	-6.286880483521	6.216602478137	-0.402703030968
H	3.029076232954	-4.046954311271	-2.784577214158
H	6.290994483830	-6.176714475085	-0.974367074924
H	4.230394325376	-3.112402239397	1.221237093921
H	6.001345461586	-4.839788372217	1.098497084469
H	4.798211369056	-5.774597444154	-2.917872224399
H	-8.861868681604	-9.123152701660	2.702197207829
H	-9.143152703209	-9.299565715247	-2.561225197014
H	-7.978754613646	-7.984753614119	-4.583367352541
H	-2.781491213947	-2.814369216459	-3.092607237833
H	-4.161711320070	-4.233149325589	-4.897399376647
H	-7.476236575031	-7.709486592951	4.506788346603
H	-3.623856278735	-3.993208307134	4.191332322342
H	-5.370295413057	-8.195856630382	-7.767592597400
H	-2.463272189478	-2.674835205712	2.169439166830
H	-7.109425546824	-4.268508328324	-7.817884601307
H	-5.242785403216	-8.102425623153	-5.297541407433
H	-6.978748536775	-4.184698321870	-5.347644411293
H	-6.305395484962	-6.277179482789	-9.037731695135
H	-6.354870488780	-3.850135296108	4.907116377424
H	-5.311795408523	-5.686811437399	8.647145665065
H	-4.661694358516	-7.786621598862	4.957129381252
H	-4.529625348401	-7.704055592551	7.427251571247
H	-6.226044478834	-3.758076289061	7.377082567396
H	-6.205349477264	-6.547759503591	0.646358049729
H	-5.413243416360	-5.415203416499	-1.036735079763
H	-16.693673283936	-13.298525022825	0.311729023975
H	-16.726167286438	-10.610308816083	0.136590010488
H	-12.984306998631	-6.864756527985	0.123683009501
H	-10.298737792105	-6.875896528825	0.105540008131
H	-15.754538211695	-8.722414670867	2.072025159343
H	-17.494221345530	-6.961359535407	1.998262153679

H	-17.869259374368	-5.672351436285	-0.090993006996
H	-16.492456268455	-6.155984473468	-2.100594161583
H	-14.752781134669	-7.917268608954	-2.015851155047
H	-13.002458000050	-16.957386304243	0.719522055323
H	-10.307958792824	-16.979946305935	0.746389057421
H	-6.566389505037	-13.259586019846	0.287153022068
H	-6.584720506467	-10.580663813771	0.117247009005
H	-8.537901656649	-16.207777246548	-1.484721114202
H	-6.795294522650	-17.962140381488	-1.342862103256
H	-5.407751415900	-18.171969397650	0.706253054340
H	-5.775146444171	-16.616385278012	2.606923200492
H	-7.518317578245	-14.864278143216	2.454684188785
H	-14.814547139426	-15.101451161478	0.439167033801

(a) 60° tilt for parallel

V	-0.232250603000	0.226360860000	-0.080930781000
V	-11.682712273000	-11.799426020000	0.930286195000
O	-0.506426332000	0.508809746000	1.449817601000
O	-11.822326165000	-11.436648028000	2.462570779000
N	-6.167369804000	-7.696045928000	-1.055650931000
N	-5.623535933000	-4.095508051000	0.737509040000
N	-7.583803200000	-6.183091131000	1.049161631000
N	-4.205150402000	-5.608904711000	-1.364380876000
N	-2.125813491000	0.053248461000	-0.927678410000
N	-0.108882982000	-1.846896334000	-0.215823883000
N	1.845335261000	0.199760178000	-0.265237396000
N	-0.179970536000	2.107906662000	-0.980025152000
N	-11.616457165000	-13.884171122000	0.864906875000
N	-9.630787273000	-11.885152205000	0.602530212000
N	-13.642995945000	-11.950253178000	0.231250458000
N	-11.647111345000	-9.962227388000	-0.044764691000
C	-3.494712482000	-8.079695200000	-4.985250802000
C	-3.552311309000	-8.532616949000	-3.662724671000
C	-2.930742887000	-9.742859606000	-3.335840243000
C	-2.266724396000	-10.484145913000	-4.309733818000
C	-2.216661582000	-10.025910166000	-5.625284334000
C	-2.833125560000	-8.821690605000	-5.960611257000
C	-4.260716659000	-7.738679093000	-2.618435704000
C	-5.426631724000	-8.275415110000	-2.043472452000
C	-7.202232536000	-8.553673971000	-0.828074022000
C	-7.120321719000	-9.716309725000	-1.700330640000
C	-6.016770192000	-9.540916571000	-2.462318362000
C	-6.364870925000	-3.515453055000	1.724670677000
C	-5.774202750000	-2.250099764000	2.144392375000
C	-4.669716696000	-2.074625117000	1.384533533000
C	-4.588110156000	-3.238283771000	0.511898553000
C	-8.236987317000	-8.373748414000	0.100034080000
C	-8.405710720000	-7.277017982000	0.945914387000
C	-8.089748510000	-5.288398456000	1.959901077000
C	-9.305362315000	-5.856146376000	2.462581363000
C	-9.494054222000	-7.064488228000	1.852155297000
C	-7.531174935000	-4.051566230000	2.299356111000
C	-8.240584664000	-3.256367874000	3.341829471000
C	-8.857758268000	-2.044345595000	3.013338989000
C	-9.522831939000	-1.301888218000	3.985510093000
C	-9.578198696000	-1.760693889000	5.300620863000

C	-8.966015233000	-2.966654972000	5.637385560000
C	-8.303428166000	-3.710011421000	4.663837537000
C	-3.550578253000	-3.419746058000	-0.413392738000
C	-3.381577561000	-4.516024043000	-1.258968239000
C	-3.701150877000	-6.502856538000	-2.277234385000
C	-2.485198927000	-5.935840999000	-2.779284950000
C	-2.294877155000	-4.729006773000	-2.165702872000
C	-2.508816108000	-2.345713626000	-0.497160438000
C	-2.928322431000	-1.062477318000	-0.838697785000
C	-2.956388934000	1.090051686000	-1.297300251000
C	-4.305706250000	0.609585203000	-1.434719116000
C	-4.287385075000	-0.719228612000	-1.154201198000
C	-1.189640198000	-2.700471407000	-0.226134372000
C	-0.765510454000	-4.037557321000	0.084795362000
C	0.577048210000	-3.991028353000	0.284880455000
C	0.986734062000	-2.625082171000	0.093143663000
C	2.302716916000	-2.175986902000	0.215413241000
C	3.360423701000	-3.170910511000	0.554432762000
C	3.757112959000	-4.135286305000	-0.377316608000
C	4.749212878000	-5.059506012000	-0.059163604000
C	5.354495340000	-5.031592441000	1.196138495000
C	4.963973114000	-4.074051501000	2.130704147000
C	3.974066106000	-3.148068046000	1.810775753000
C	-2.570593848000	2.415540151000	-1.503945025000
C	-3.614848418000	3.400986724000	-1.906465817000
C	-4.029915472000	4.393788332000	-1.013422587000
C	-5.003440741000	5.317433353000	-1.386081377000
C	-5.570630379000	5.261223617000	-2.658064201000
C	-5.160593683000	4.276029461000	-3.554836014000
C	-4.189363457000	3.350723353000	-3.180417709000
C	2.690209073000	-0.848602924000	0.022899616000
C	4.052896582000	-0.384203321000	0.060060559000
C	4.024733240000	0.950322433000	-0.198537249000
C	2.649929688000	1.307591691000	-0.403596609000
C	2.216937813000	2.582206642000	-0.731664807000
C	0.910903491000	2.945991130000	-1.017511540000
C	-1.257603491000	2.870891353000	-1.370492344000
C	-0.827405142000	4.213771228000	-1.662928392000
C	0.512846839000	4.259010062000	-1.438597985000
C	-14.027345319000	-14.327574560000	0.711399435000
C	-12.715353520000	-14.709835271000	0.939886000000
C	-12.308929517000	-16.047756756000	1.262551827000
C	-10.954549033000	-16.032868354000	1.381422500000
C	-10.524399897000	-14.683084303000	1.122786061000
C	-9.193729729000	-14.261210442000	1.127867221000
C	-8.794824806000	-12.950730502000	0.858785882000
C	-7.422953955000	-12.521576220000	0.792740328000
C	-7.433107808000	-11.191414061000	0.517790942000
C	-8.810186292000	-10.798702628000	0.399131411000
C	-8.134946068000	-15.269138581000	1.422612599000
C	-7.831321625000	-16.274366858000	0.499107810000
C	-6.838967701000	-17.211766254000	0.775327221000
C	-6.140143635000	-17.156199468000	1.980102948000
C	-6.437505794000	-16.157801811000	2.906200705000
C	-7.428018710000	-15.219071005000	2.628152986000
C	-14.450957528000	-13.057005169000	0.355915092000

C	-15.808240549000	-12.718301566000	0.035470222000
C	-15.820666690000	-11.398130803000	-0.289915048000
C	-14.468643161000	-10.919858818000	-0.158173726000
C	-14.065642406000	-9.607996138000	-0.415439066000
C	-12.738687590000	-9.178931816000	-0.356497717000
C	-12.313324334000	-7.828309548000	-0.612015185000
C	-10.963826568000	-7.798645988000	-0.461631477000
C	-10.551705972000	-9.129616730000	-0.111107643000
C	-15.113549357000	-8.614348905000	-0.786682836000
C	-16.036855705000	-8.172550991000	0.166203461000
C	-17.018224438000	-7.245873784000	-0.177093400000
C	-17.089975052000	-6.752763669000	-1.478856203000
C	-16.174784696000	-7.189934986000	-2.435046154000
C	-15.191614114000	-8.114144110000	-2.090286647000
C	-9.227737304000	-9.503059231000	0.104499277000
H	2.972026650000	3.356669717000	-0.803798016000
H	4.856394554000	1.637776598000	-0.264799766000
H	1.184941634000	5.096778082000	-1.561404679000
H	-1.470749737000	5.007193666000	-2.009405609000
H	-5.115661772000	-1.410683356000	-1.156609484000
H	-5.154300708000	1.216493827000	-1.707904225000
H	4.913470554000	-1.007742398000	0.244616417000
H	1.236149058000	-4.804931538000	0.542163788000
H	-5.320444901000	6.079255971000	-0.681342696000
H	-1.416955694000	-4.895692844000	0.142538125000
H	-5.594914852000	4.228646401000	-4.548167857000
H	-3.590351745000	4.433642788000	-0.022486329000
H	-3.866277992000	2.586297712000	-3.879098369000
H	-6.328335998000	5.981399456000	-2.949021728000
H	3.288470412000	-4.153366704000	-1.355540970000
H	6.126418364000	-5.752513362000	1.444802059000
H	3.666051608000	-2.404777744000	2.538379124000
H	5.427976364000	-4.048754712000	3.111308748000
H	5.050889948000	-5.799693747000	-0.793122394000
H	-10.314475812000	-7.750686125000	1.994717765000
H	-7.820629599000	-10.537706059000	-1.722388335000
H	-5.638442856000	-10.187133540000	-3.239130454000
H	-1.474781009000	-4.042496132000	-2.306255524000
H	-1.843907011000	-6.409070983000	-3.505326719000
H	-9.948369295000	-5.382525859000	3.186751961000
H	-6.152534320000	-1.603774313000	2.920979567000
H	-1.785705452000	-11.418701510000	-4.039811616000
H	-3.969733731000	-1.252701193000	1.409272041000
H	-2.802811112000	-8.460410714000	-6.983455845000
H	-2.965388122000	-10.096978671000	-2.311187456000
H	-3.980897784000	-7.146266221000	-5.248119071000
H	-1.700437005000	-10.604123625000	-6.384641516000
H	-8.818957934000	-1.689691532000	1.989046043000
H	-10.095158301000	-1.181416784000	6.058648751000
H	-7.820787823000	-4.644924742000	4.927859413000
H	-9.000608079000	-3.328419590000	6.659894649000
H	-10.000398531000	-0.365901726000	3.714540120000
H	-6.729417427000	-6.040064575000	0.526384781000
H	-5.059667940000	-5.750784540000	-0.841347375000
H	-12.982393822000	-16.885460216000	1.378109322000
H	-10.299436065000	-16.856006740000	1.619289261000

H	-6.566754162000	-13.163343337000	0.927959932000
H	-6.588609711000	-10.532737085000	0.386669816000
H	-8.371915873000	-16.313502691000	-0.440626316000
H	-6.609696366000	-17.983444799000	0.047625952000
H	-5.367444307000	-17.886817033000	2.195873912000
H	-5.899793974000	-16.110103361000	3.847533475000
H	-7.662759621000	-14.443297158000	3.349143229000
H	-16.635500697000	-13.414151656000	0.044162487000
H	-16.660559356000	-10.798589385000	-0.603858447000
H	-12.968064232000	-7.011517056000	-0.871286636000
H	-10.299930846000	-6.956472568000	-0.580373560000
H	-15.977067671000	-8.554130121000	1.179900162000
H	-17.724826352000	-6.907120439000	0.573565695000
H	-17.855109261000	-6.031537413000	-1.746865958000
H	-16.226972156000	-6.813328005000	-3.451419433000
H	-14.481543973000	-8.458172294000	-2.834795114000
H	-14.786618890000	-15.097365529000	0.789439849000

3. W-band Echo-detected EPR

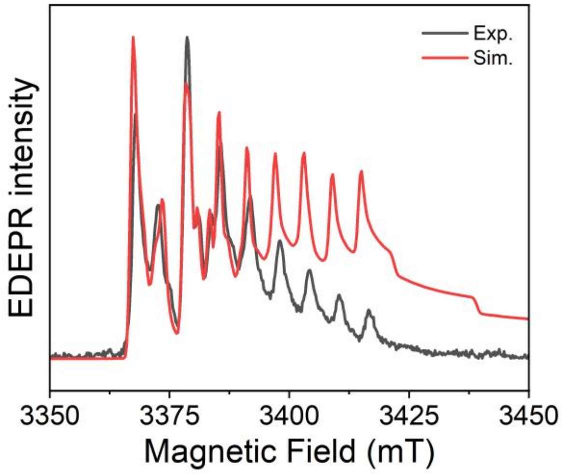

Figure S10. W-band echo-detected EPR spectrum of **VO-FP-VO** (black trace) in toluene at 85 K, overlaid with the spectral simulation (red trace) performed using the same EPR parameters of the VO monomer: **g** = [1.985, 1.985, 1.964] and **A**(^{51}V) = [162, 162, 475].[4, 14]

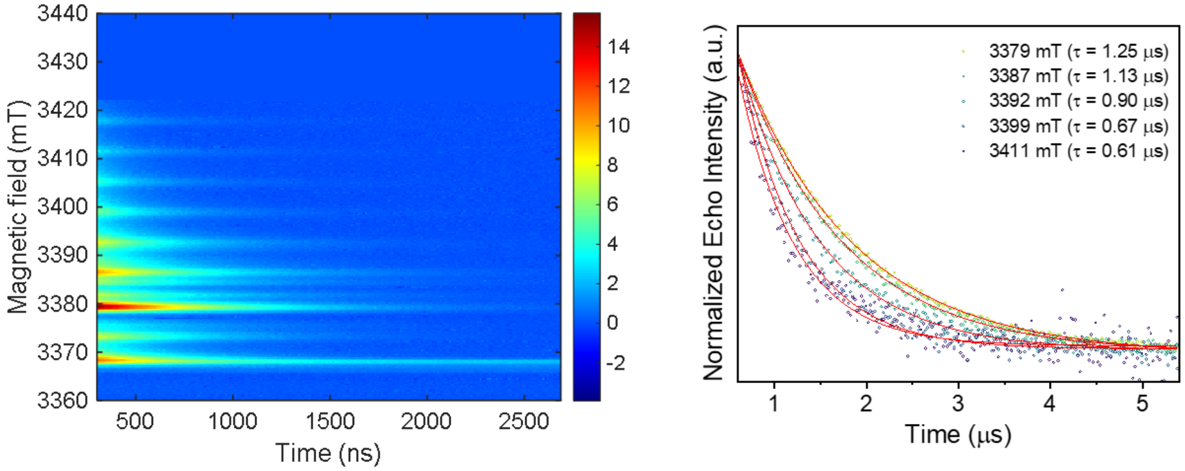

Figure S11. (a) W-band echo decay traces as a function of magnetic field for **VOFPVO** in toluene at 85 K. (b) Best fits of the echo decay traces at selected magnetic field positions.

4. Transient absorption spectroscopy

	λ_{ex}/nm	ISC time (ps)	Trip. Rec. time (ns)
**VO**	540	<0.3	74 ± 3
**VO-FP**	640	7.7 ± 0.7	$(46.3 \pm 0.4)\times10^{3}$
	545	1.1 ± 0.3	$(35 \pm 1)\times10^{3}$
**VO-FP-VO**	550	0.7 ± 0.3	$(20.3 \pm 0.1)\times10^{3}$

Table S5. Comparison of kinetic time constants for enhanced intersystem crossing (ISC) and triplet-state decay, obtained from global analysis of fs/nsTA spectra recorded at room temperature in toluene for **VO**, **VO-FP**, and **VO-FP-VO**. Data for **VO** and **VO-FP** are taken from our previous publication.[17]

5. Time-resolved electron paramagnetic resonance

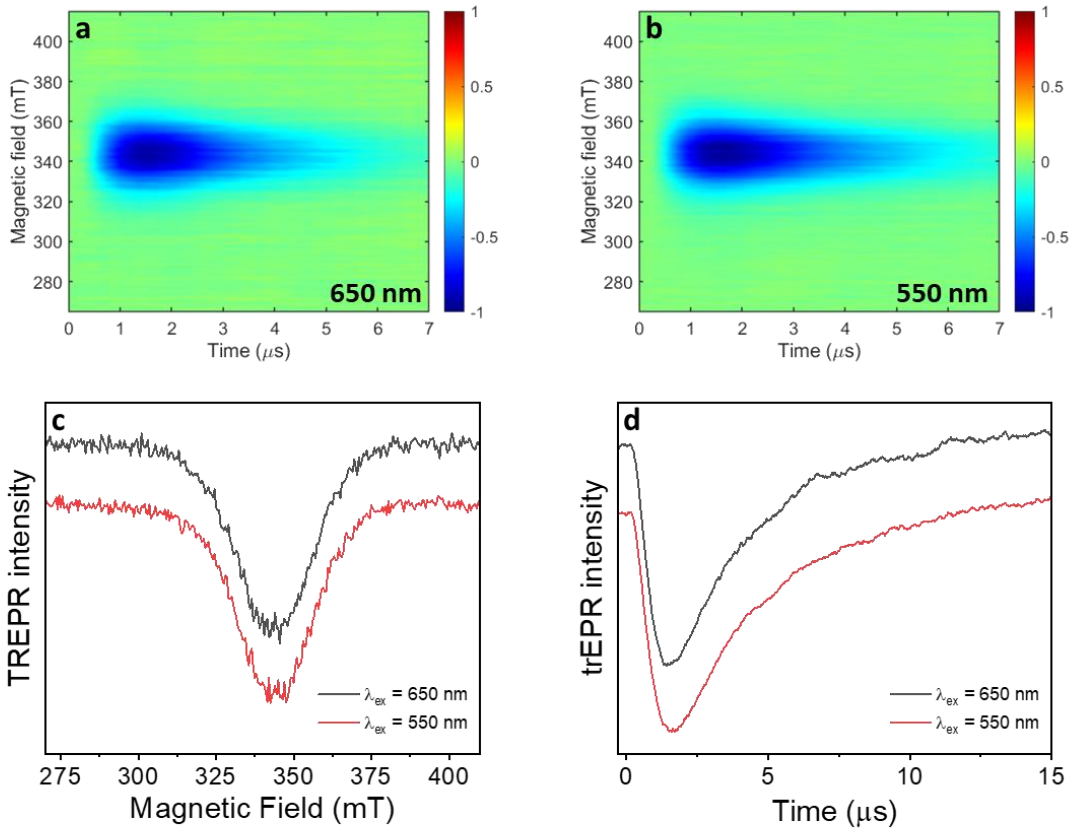

Figure S12. Comparison of normalized experimental TREPR data for **VO-FP-VO** in toluene at 85 K following excitation at 550 nm and 650 nm. (a, b) Normalized 2D TREPR contour plots. (c) 1D TREPR pulse (Δt = 200 ns). (d) 1D TREPR transients recorded at 345 mT (ΔB = 1 mT). The comparison shows no significant differences between the two excitation wavelengths, consistent with the TA analysis at RT.

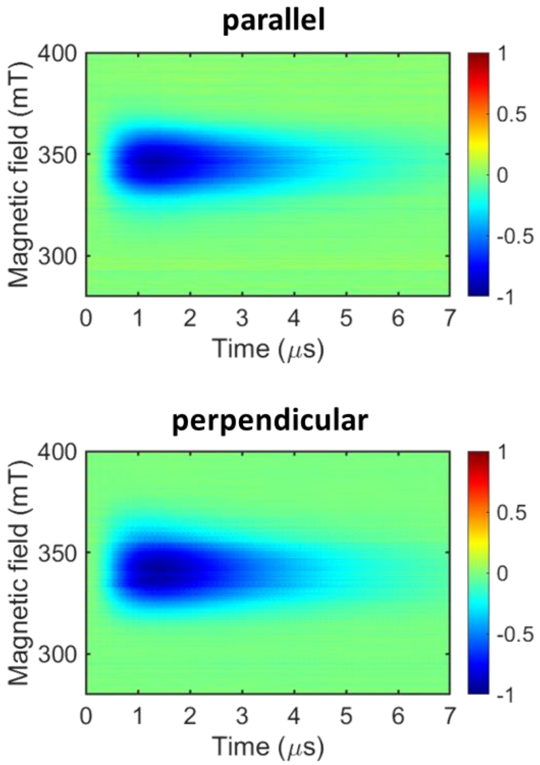

Figure S13. Normalized 2D experimental TREPR contour plots of **VO-FP-VO** oriented in the nematic liquid crystal 5CB at 85 K acquired after a 550-nm laser pulse (7 ns, 2 mJ). The long axis y of each molecule is aligned at 0° (parallel) and 90° (perpendicular) relative to the applied magnetic field direction. Color legend: red = enhanced absorption, blue = emission, green = baseline.

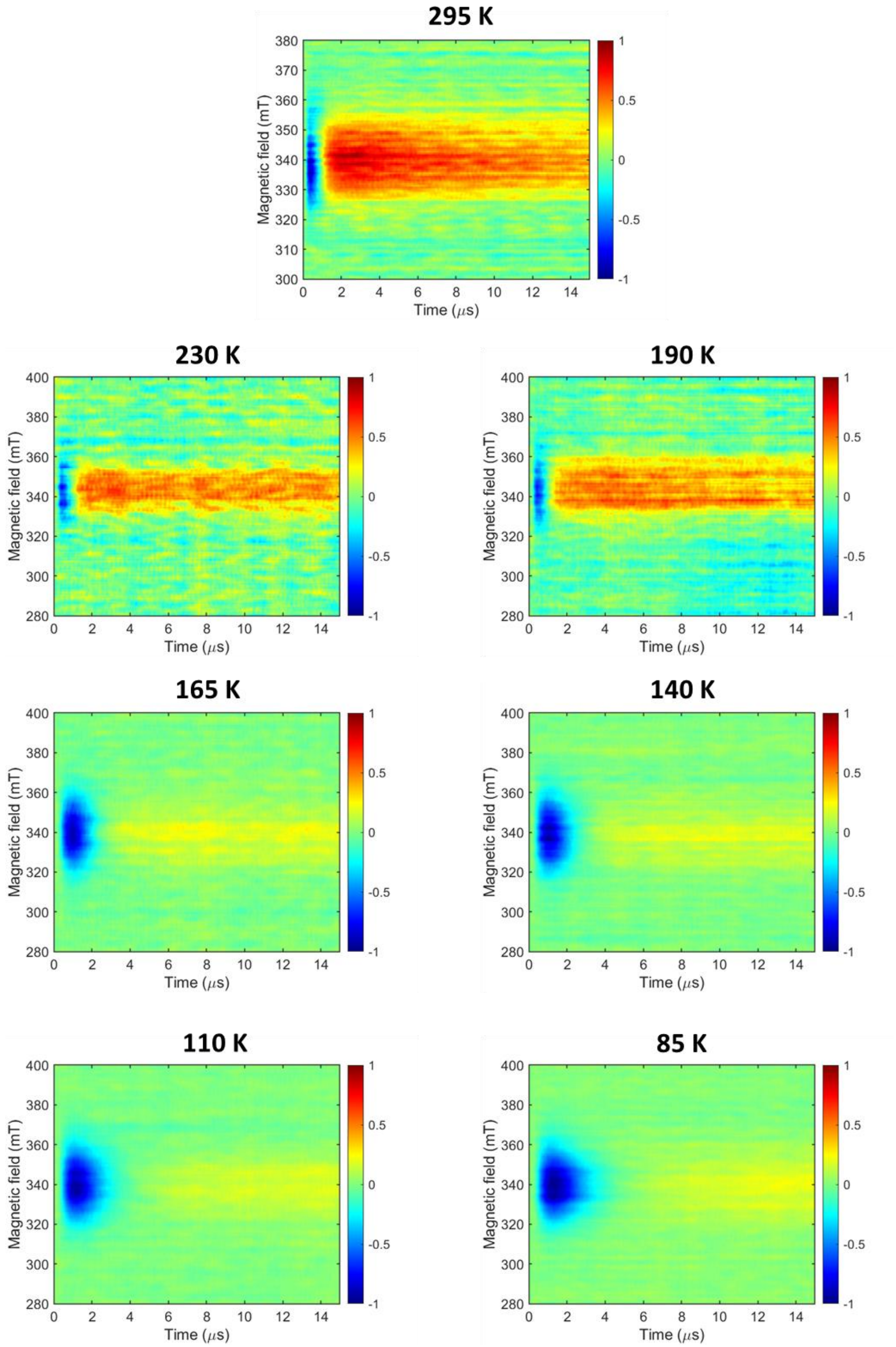

Figure S14. Normalized 2D experimental TREPR contour plots of **VO-FP-VO** in toluene solution acquired after a 550-nm laser pulse (7 ns, 2 mJ). The spectra are acquired at seven different temperatures. Color legend: red = enhanced absorption, blue = emission, green = baseline.

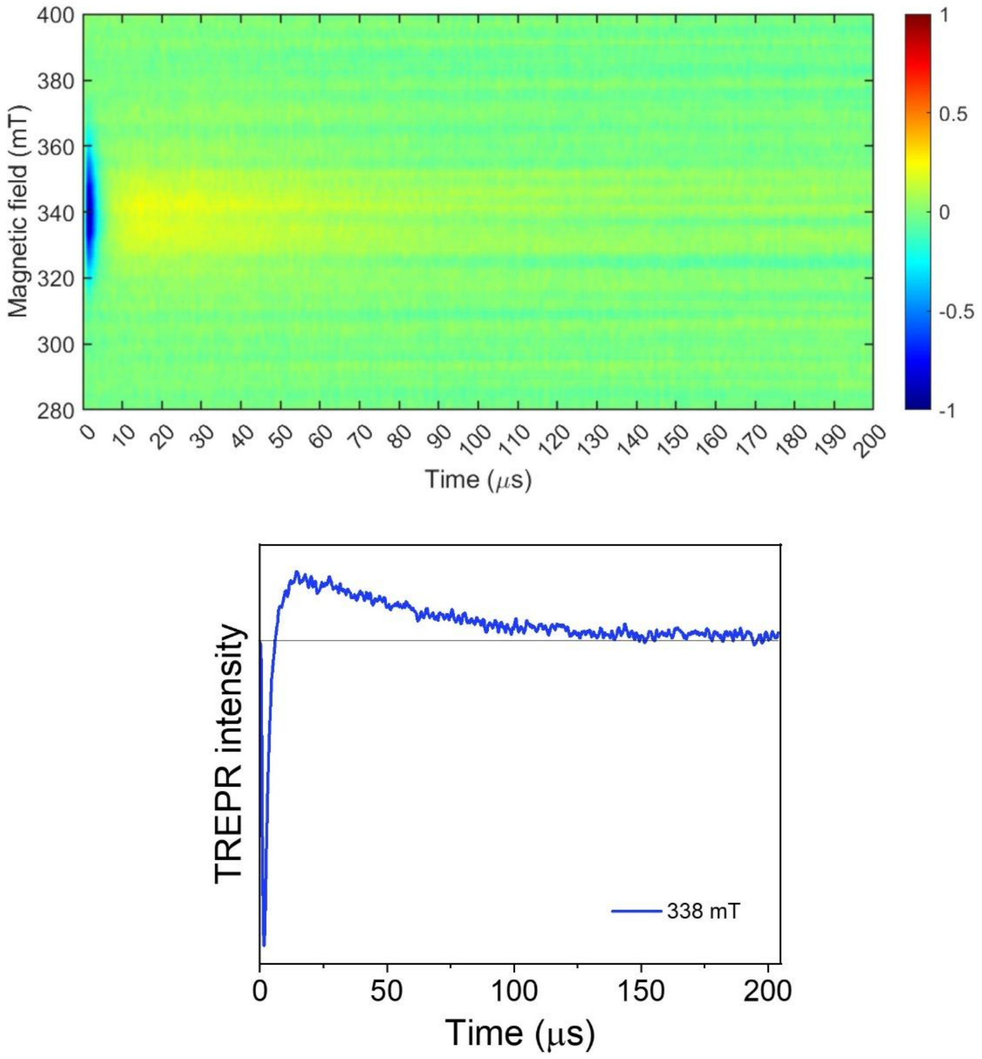

Figure S15. (Top) Normalized 2D experimental TREPR contour plot of **VO-FP-VO** in frozen toluene at 85 K, recorded over 200 μs following a 550-nm laser pulse (7 ns, 2 mJ). Color legend: red = enhanced absorption, blue = emission, green = baseline. (Bottom) TREPR transient acquired at 338 mT (integration window = 20 mT), showing the characteristic evolution of spin polarization: initial emission at short times followed by absorption at later times. The signal decays to zero after approximately 100 μs.

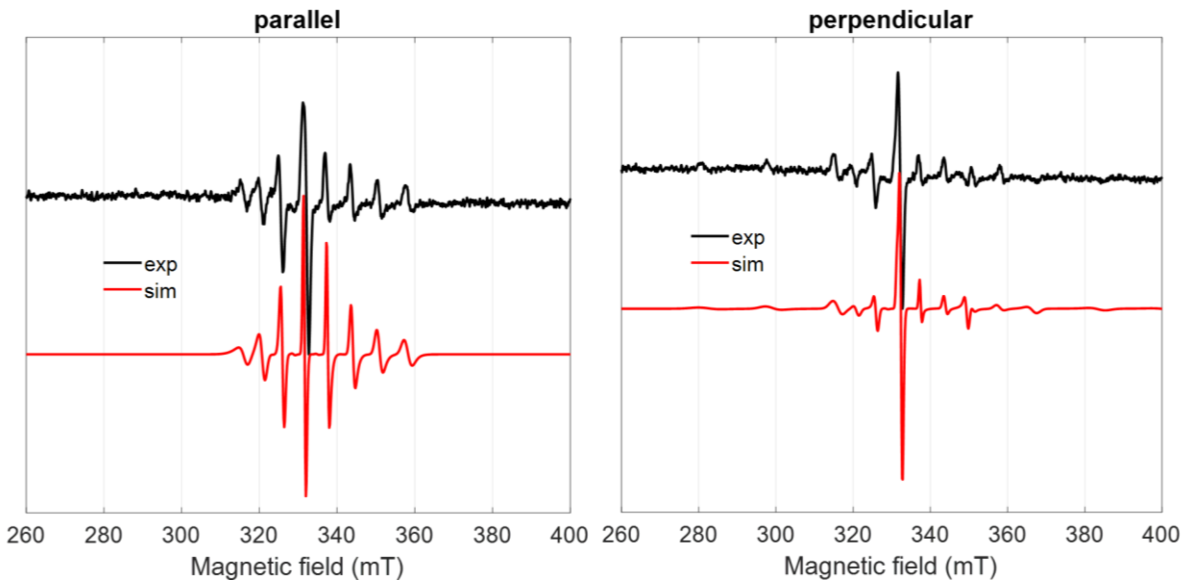

Figure S16. Dark CWEPR spectrum (black) and corresponding spectral simulations (red) of VO–FP–VO aligned in the nematic liquid crystal 5CB at 85 K. The molecules are oriented with their long axis parallel (a) and perpendicular (b) to the external magnetic field direction, with a gaussian distribution of width σ=5∘ and a strain in the hyperfine tensor of 10%.

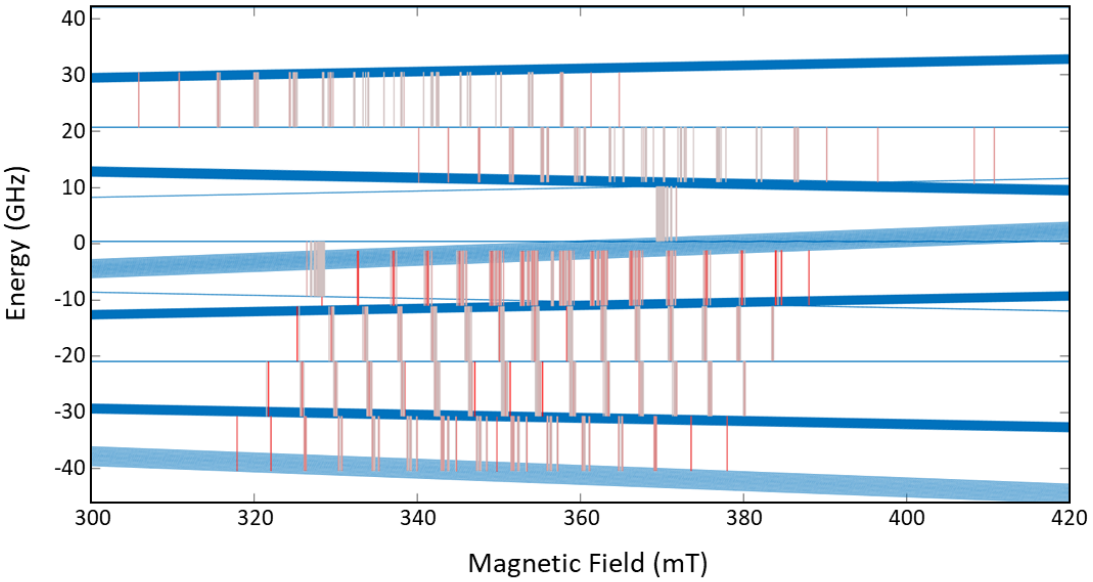

Figure S17. Zeeman energy-level diagram and transitions of **VO-FP-VO** for $z \parallel \boldsymbol{B_0}$. The color scale represents the transition amplitude, with red indicating allowed resonances and grey forbidden ones; transitions with relative intensity below 0.05 are omitted.

^{2}**VO**	**g**	[1.985 1.985 1.964]
	**A**/MHz	[162 162 475]
^{3}**FP**	**g**	2.002
	**D**/MHz	[1153 -224]
	**d**/MHz	87.7*[1 -2 1]
	J/cm^{-1}	-0.7
	ϑ	80°
	[p_1 p_2 p_3 p_4 p_5]	[0, 0.09, 0.25, 0.33, 0.33]

Table S6. Parameters used for spectral simulations of the TREPR spectra of **VO–FP–VO** in 5CB, shown in Figures 3a and 3b. The **A** and **g** tensors of VO, as well as the g-value and zero-field splitting tensor **D** of the ^{3}FP state, were fixed based on literature values. The magnetic dipole-dipole interaction was calculated within the point-dipole approximation using the distances between spin centers from DFT-optimized molecular structure. The exchange interaction J was calculated from broken-symmetry DFT calculations. ϑ is the dihedral angle between the z and z' axes, corresponding to the local easy axes of the VO and FP units, respectively. Variations of J and θ within the range of configurations accessible to the system do not significantly affect the simulated spectra. The five quintet spin-state populations, expressed in the basis of the Hamiltonian eigenstates in the applied magnetic field and ordered from $p_1$to $p_5$with increasing energy, were fitted to obtain the best agreement with the experimental spectrum.

6. Derivation of the Hamiltonian for the $\sqrt{iSWAP}$ gate

We outline here a derivation of the effective Hamiltonian H_{qq} given in the main text. We consider the situation with the switch in the "on state", i.e., we start from a three-spin Hamiltonian with an $S = 1$ interposed between the two qubits and linked to them by an isotropic exchange interaction. For the sake of simplicity, we neglect, for the moment, zero-field splitting terms on the switch. These do not qualitatively affect our conclusions, but they make the expressions less clear.

The corresponding spin Hamiltonian is

$$H = H_0 + H_1 = g_1\mu_B B(s_{z1} + s_{z3}) + g_2\mu_B B S_{z2} + J\mathbf{S}_2 \cdot (\mathbf{s}_1 + \mathbf{s}_3)$$

where we have separated the leading Zeeman interaction terms in H_0 from the exchange coupling H_1. Our scheme relies on a perturbative picture in which $J \ll |g_2 - g_1|\mu_B B$. Hence, we can start from the eigenstates of H_0 ($|m_1 m_2 m_3\rangle$ such that $H_0|m_1 m_2 m_3\rangle = E_{m_1 m_2 m_3}|m_1 m_2 m_3\rangle$) and treat H_1 as a perturbation. In particular, we consider the effect of H_1 within a subspace of the eigenstates of H_0 with fixed m_{FP}. This subspace forms our computational basis (four states corresponding to the different eigenstates of s_{z1} and s_{z3}).

Let us start from the subspace with $m_{FP} = -1$, i.e. from the set of eigenstates of H_0 $|\uparrow, -1, \uparrow\rangle$, $|\uparrow, -1, \downarrow\rangle, |\downarrow, -1, \uparrow\rangle, |\downarrow, -1, \downarrow\rangle$, usually labeled in the effective 4-state Hamiltonian H_{qq} as $|00\rangle, |01\rangle, |10\rangle, |11\rangle$. We now compute the matrix elements of H_{qq} by considering H_1 as a perturbation.

The diagonal part of H_1, namely $JS_{z2}(s_{z1} + s_{z3})$, provides a first order correction which only renormalizes the Zeeman energies, i.e. $\langle 00|JS_z(s_{z1} + s_{z3})|00\rangle = -\langle 11|JS_z(s_{z1} + s_{z3})|11\rangle = -J$ and $\langle 01|JS_z(s_{z1} + s_{z3})|01\rangle = \langle 10|JS_z(s_{z1} + s_{z3})|10\rangle = 0$. Conversely, the transverse part of the

exchange interaction, $J[S_{x2}(s_{x1}+s_{x3})+S_{y2}(s_{y1}+s_{y3})]$ couples states with different m_{FP}, yielding second order contributions to H_{qq}. For instance, the only off-diagonal contributions to H_{qq} are those connecting $|01\rangle$ and $|10\rangle$, of the form

$$\langle 01|H_{qq}|10\rangle = \frac{\langle\uparrow,-1,\downarrow|H_1|\downarrow,0,\downarrow\rangle\langle\downarrow,0,\downarrow|H_1|\downarrow,-1,\uparrow\rangle}{E_{\frac{1}{2},-1,-\frac{1}{2}} - E_{-\frac{1}{2},0,-\frac{1}{2}}} = \frac{J^2}{(g_1-g_2)\mu_B B}$$

By including also diagonal second-order corrections we obtain the full H_{qq} matrix (apart from a contribution proportional to the identity in this subspace):

$$\begin{pmatrix} g_1\mu_B B - J + \frac{2J^2}{(g_1-g_2)\mu_B B} & 0 & 0 & 0 \\ 0 & \frac{J^2}{(g_1-g_2)\mu_B B} & \frac{J^2}{(g_1-g_2)\mu_B B} & 0 \\ 0 & \frac{J^2}{(g_1-g_2)\mu_B B} & \frac{J^2}{(g_1-g_2)\mu_B B} & 0 \\ 0 & 0 & 0 & -g_1\mu_B B + J \end{pmatrix}$$

This can be recast in terms of spin 1/2 operators as reported in the main text, i.e. an XY coupling $\frac{J^2}{(g_1-g_2)\mu_B B}$ and an effective Zeeman interaction $g_1\mu_B B - J + \frac{J^2}{(g_1-g_2)\mu_B B}$.

We can derive an equivalent effective Hamiltonian also for the $m_{FP}=1$ subspace. In that case the XY coupling and the first order contribution to the effective Zeeman term change sign.

Note that the inclusion of additional terms in H_0 (such as the zero field splitting on S_2) only changes the matrix elements and the energy differences appearing in the denominators of the above equation, but it does not alter the overall description. The effective couplings in the $m_{FP}=-1$ and $m_{FP}=1$ subspaces will become different, but for sufficiently large magnetic field (e.g. by

working in W band) we can make them similar and operate also with a mixture of the two (see discussion on this point in the main text).

7. References

1. Brückner, C.; Posakony, J. J.; Johnson, C. K.; Boyle, R. W.; James, B. R.; Dolphin, D., Novel and improved syntheses of 5,15-diphenylporphyrin and its dipyrrolic precursors. *J. Porphyr. Phthalocya.* **1998,** *2* (6), 455-465.

2. Boyle, R. W.; Bruckner, C.; Posakony, J.; James, B. R.; Dolphin, D., 5-Phenyldipyrromethane and 5, 15-Diphenylporphyrin. *Organic syntheses* **2003,** *76*, 287-287.

3. Yamabayashi, T.; Atzori, M.; Tesi, L.; Cosquer, G.; Santanni, F.; Boulon, M.-E.; Morra, E.; Benci, S.; Torre, R.; Chiesa, M.; Sorace, L.; Sessoli, R.; Yamashita, M., Scaling Up Electronic Spin Qubits into a Three-Dimensional Metal–Organic Framework. *J. Am. Chem. Soc.* **2018,** *140* (38), 12090-12101.

4. Ranieri, D.; Privitera, A.; Santanni, F.; Urbanska, K.; Strachan, G. J.; Twamley, B.; Salvadori, E.; Liao, Y.-K.; Chiesa, M.; Senge, M. O.; Totti, F.; Sorace, L.; Sessoli, R., A Heterometallic Porphyrin Dimer as a Potential Quantum Gate: Magneto-Structural Correlations and Spin Coherence Properties. *Angew. Chem. Int. Ed.* **2023,** *62*, e202312936.

5. Marques, A. T.; Pinto, S. M. A.; Monteiro, C. J. P.; Seixas de Melo, J. S.; Burrows, H. D.; Scherf, U.; Calvete, M. J. F.; Pereira, M. M., Energy transfer from fluorene-based conjugated polyelectrolytes to on-chain and self-assembled porphyrin units. *J. Polym. Sci., Part A: Polym. Chem.* **2012,** *50* (7), 1408-1417.

6. Bakar, M. A.; Sergeeva, N. N.; Juillard, T.; Senge, M. O., Synthesis of Ferrocenyl Porphyrins via Suzuki Coupling and Their Photophysical Properties. *Organometallics* **2011,** *30* (11), 3225-3228.

7. Neese, F., Software update: The ORCA program system—version 6.0. *Wiley Interdisciplinary Reviews: Computational Molecular Science* **2025,** *15* (2), e70019.

8. Becke, A. D., Density-functional thermochemistry. III. The role of exact exchange. *J. Chem. Phys.* **1993,** *98* (7), 5648-5652.

9. Lee, C.; Yang, W.; Parr, R. G., Development of the Colle-Salvetti correlation-energy formula into a functional of the electron density. *Phys. Rev. B* **1988,** *37* (2), 785-789.

10. Stoychev, G. L.; Auer, A. A.; Neese, F., Automatic Generation of Auxiliary Basis Sets. *J. Chem. Theory Comput.* **2017,** *13* (2), 554-562.

11. Grimme, S.; Antony, J.; Ehrlich, S.; Krieg, H., A consistent and accurate ab initio parametrization of density functional dispersion correction (DFT-D) for the 94 elements H-Pu. *J. Chem. Phys.* **2010,** *132* (15), 154104

12. Grimme, S.; Ehrlich, S.; Goerigk, L., Effect of the damping function in dispersion corrected density functional theory. *J. Comput. Chem.* **2011,** *32* (7), 1456-1465.

13. Barone, V.; Cossi, M., Quantum Calculation of Molecular Energies and Energy Gradients in Solution by a Conductor Solvent Model. *J. Phys. Chem. A* **1998,** *102* (11), 1995-2001.

14. Ranieri, D.; Santanni, F.; Privitera, A.; Albino, A.; Salvadori, E.; Chiesa, M.; Totti, F.; Sorace, L.; Sessoli, R., An exchange coupled meso–meso linked vanadyl porphyrin dimer for quantum information processing. *Chem. Sci.* **2023,** *14* (1), 61-69.

15. Ahmed, N.; Sahu, P. P.; Chakraborty, A.; Flores Gonzalez, J.; Ali, J.; Kalita, P.; Pointillart, F.; Singh, S. K.; Chandrasekhar, V., In situ hydrolysis of a carbophosphazene ligand leads to one-dimensional lanthanide coordination polymers. Synthesis, structure and dynamic magnetic studies. *Dalton Trans.* **2024,** *53* (27), 11563-11577.

16. Yanai, T.; Tew, D. P.; Handy, N. C., A new hybrid exchange–correlation functional using the Coulomb-attenuating method (CAM-B3LYP). *Chem. Phys. Lett.* **2004,** *393* (1), 51-57.

17. Privitera, A.; Chiesa, A.; Santanni, F.; Carella, A.; Ranieri, D.; Caneschi, A.; Krzyaniak, M. D.; Young, R. M.; Wasielewski, M. R.; Carretta, S.; Sessoli, R., Room-Temperature Optical Spin Polarization of an Electron Spin Qudit in a Vanadyl-Free Base Porphyrin Dimer. *J. Am. Chem. Soc.* **2025,** *147* (1), 331-341.

18. Stoll, S.; Schweiger, A., EasySpin, a comprehensive software package for spectral simulation and analysis in EPR. *J. Magn. Reson.* **2006,** *178* (1), 42-55.
